# NAMPT and NAPRT: Two Metabolic Enzymes With Key Roles in Inflammation

**DOI:** 10.3389/fonc.2020.00358

**Published:** 2020-03-19

**Authors:** Valentina Audrito, Vincenzo Gianluca Messana, Silvia Deaglio

**Affiliations:** Laboratory of Tumor Immunogenetics, Department of Medical Sciences, University of Turin, Turin, Italy

**Keywords:** inflammation, cancer, signaling, metabolism, DAMPs, NAMPT, NAPRT, TLRs

## Abstract

Nicotinamide phosphoribosyltransferase (NAMPT) and nicotinate phosphoribosyltransferase (NAPRT) are two intracellular enzymes that catalyze the first step in the biosynthesis of NAD from nicotinamide and nicotinic acid, respectively. By fine tuning intracellular NAD levels, they are involved in the regulation/reprogramming of cellular metabolism and in the control of the activity of NAD-dependent enzymes, including sirtuins, PARPs, and NADases. However, during evolution they both acquired novel functions as extracellular endogenous mediators of inflammation. It is well-known that cellular stress and/or damage induce release in the extracellular milieu of endogenous molecules, called alarmins or damage-associated molecular patterns (DAMPs), which modulate immune functions through binding pattern recognition receptors (PRRs), such as Toll-like receptors (TLRs), and activate inflammatory responses. Increasing evidence suggests that extracellular (e)NAMPT and eNAPRT are novel soluble factors with cytokine/adipokine/DAMP-like actions. Elevated eNAMPT were reported in several metabolic and inflammatory disorders, including obesity, diabetes, and cancer, while eNAPRT is emerging as a biomarker of sepsis and septic shock. This review will discuss available data concerning the dual role of this unique family of enzymes.

## Introduction

One of the key roles of the innate immune system is to initiate immune responses against invasive pathogens. Pathogen-associated molecular patterns (PAMPs) include sugars/lipoproteins or nucleic acids [i.e., bacterial DNA as unmethylated repeats of dinucleotide CpG, double-stranded (ds) or single-stranded (ss) RNA] (1, 2). PAMPs can initiate immune responses through the activation of classical pattern recognition receptors (PRRs), among which there are toll-like receptors (TLRs), NOD-like receptors (NLRs), retinoic acid inducible gene I (RIG- I)-like receptors (RLRs), C-type lectin receptors (CLRs), multiple intracellular DNA sensors, and other non-PRRs DAMPs receptors (2–4). However, these receptors can be engaged also by endogenous ligands. It is now largely accepted that cells in conditions of hypoxia, acidosis, redox imbalance, hypertonic/hypotonic stress, and intracellular ion or cytoskeleton perturbations, can release small endogenous molecules called damage-associated molecular patterns (DAMPs) or sometimes “danger signals” or “alarmins,” triggering immune responses through the activation of PRRs (4–6). Intriguingly, many of these DAMPs have a well-characterized intracellular function and have been serendipitously identified in the extracellular space where they initiate inflammatory responses, independently of pathogen infection, a phenomenon referred to as sterile inflammation (4, 7, 8). Similar to pathogen-induced inflammation, DAMPs can prime neutrophils, macrophages, and dendritic cells (DCs), but also non-immune cells, including endothelial and epithelial cells and fibroblasts (7). Activation of these cells leads to the production of several cytokines and chemokines, which in turn recruit inflammatory elements and trigger adaptive immune responses. Although sterile inflammation plays an essential role in tissue repair and regeneration, unresolved chronic inflammation is deleterious to the host leading to the development of metabolic, neurodegenerative, autoimmune disorders, and cancer (4).

Since their original definition as DAMPs in 2003, the list of endogenous molecules are increased considerably (4) and now includes high-mobility group box 1 protein (HMGB-1), heat shock proteins (HSPs), histone and extracellular matrix components (for example, hyaluronic acid and biglycan). All these molecules exert pro-inflammatory functions through binding to TLRs. HMGB-1 is among the most studied DAMPs. It is a nuclear DNA binding protein that can be found in the extracellular space not only as a consequence of necrosis, but also through dedicated secretion mechanisms (9, 10). Extracellularly, HMGB-1 elicits pro-inflammatory effects linked to consequent TLR4 binding and activation of the nuclear-factor kappa B (NF-kB) signaling pathway (11, 12). In animal models, HMGB-1 is as a late mediator of lethal systemic inflammation, involved in delayed endotoxin lethality (13). Others DAMPs include F-actin, Sin3A associated protein 130 (SAP130), β-glucosylceramide and N-glycans binding to CLRs; monosodium urate (MSU) crystals, cholesterol crystals, β-amyloid (Aβ), and adenosine 5′-triphosphate (ATP) that activate NLRP3 inflammasome (4). In addition, numerous cytokines [i.e., interleukin (IL)-1β, tumor necrosis factor (TNF), and type I interferon (IFN-I)], pro-inflammatory proteins, such as interferon-induced protein 35, and bioactive lipids like lysophospholipids, are referred as “inducible DAMPs” or “conditional DAMPs” (14).

Nucleotides and nucleosides, for long time considered simply electron-shuttling agents involved in supporting energy metabolism, are gaining interest together with the network of enzymes that control their synthesis and degradation. Interestingly, while all these factors have a well-characterized intracellular function, they can be released in the extracellular space, where they bind and activate different sets of cellular receptors, including purinergic and PRRs. For example, ATP a key intracellular energy molecule, can be massively released by passive leakage when cells become injured, stressed, or even necrotic, acting as a DAMP (15). Extracellular ATP and its derivative nucleotides (adenosine, AMP, ADP) synthesized by endonucleotidases achieve many of their effects through purinergic receptors, via inflammatory cascades and the production of proinflammatory cytokines (16, 17). Among the enzymes involved in nicotinamide adenine dinucleotide (NAD) synthesis, nicotinamide phosphoribosyltransferase (NAMPT)—the focus of this review—emerges as new mediator of inflammation. Intracellularly, it catalyzes the first and rate-limiting step in the biosynthesis of NAD from nicotinamide (Nam) (18, 19). Increased eNAMPT levels are reported in conditions of acute or chronic inflammation (18, 20–25). eNAMPT effects are mostly linked to the activation of an inflammatory signature mainly in macrophages, with recent data suggesting that it binds TLR4, therefore adding the enzyme to the number of “danger” signals activating this receptor (26). NAMPT is structurally and functionally related to a second NAD-biosynthetic enzyme (NBE), i.e., nicotinate phosphoribosyltransferase (NAPRT), which is rate-limiting in the NAD salvage pathway that starts form nicotinic acid (Na) (27–29). Our group recently discovered the presence of NAPRT in extracellular fluids (eNAPRT), highlighting a role also for this enzyme as a ligand for TLR4.

This review summarizes the current knowledge on NAMPT and NAPRT, as intracellular NBEs involved in the regulation/reprogramming of cellular metabolism, and as cytokines/DAMPs in the extracellular environment. Lastly, we will discuss the role of these enzymes especially in relation to the development of inflammatory conditions, including cancer, and their potential therapeutic values.

## NAD Levels Modulate Cellular Transcriptional Responses and Metabolic Adaptation

**Figure 1:**
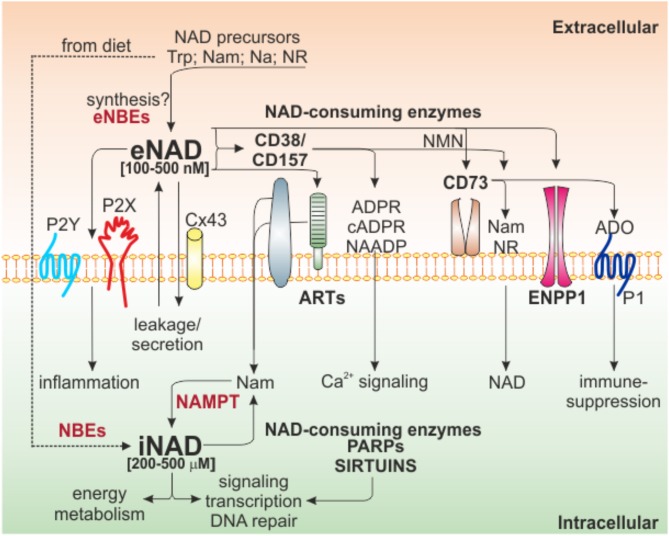
Intra/extra NAD interconnections and NAD-metabolizing enzymes. Schematic representation of the network of NAD-metabolizing cell surface and intracellular enzymes and their products. Several NAD precursors derived from diet can be internalized to generate iNAD, via NBE activities, to support energy metabolism, signaling, and other biological processes through the activities of intracellular NAD-consuming enzymes (PARPs and Sirtuins). These enzymes release Nam that, in turn, via NAMPT-dependent salvage pathway, support NAD production. Once in the extracellular space because of secretion/leakage, via Cx43, or because of direct extracellular synthesis from precursors (not confirmed), eNAD can function by binding purinergic receptors (P2Y, P2X), an event that leads to intracellular signaling and inflammatory conditions. Alternatively, eNAD can also be metabolized by a series of ecto-enzymes of the cell surface (CD38/CD157, ARTs, CD73, ENPP1) generating different metabolites (cADPR, ADPR, and NAADP) involved mainly in Ca^2+^-signaling. The end product of the reaction, adenosine, can modify signal transduction by acting on P1 purinergic receptors, generally leading to immunosuppression. In the square brackets are indicated the range of iNAD [200–500 μM] or eNAD [100–500 nM]. Trp, tryptophan; Nam, nicotinamide; NR, nicotinamide riboside; Na, nicotinic acid; NBEs, NAD-biosynthetic enzymes; NAMPT, nicotinamide phosphoribosyltransferase; ARTs, mono adenosine diphosphate (ADP)-ribose transferases; PARPs, poly ADP-ribose polymerases; Cx43, connexin 43; ADPR, ADP ribose; cADPR, cyclic ADP ribose; NAADP, nicotinic acid adenine dinucleotide phosphate; NMN, nicotinamide mononucleotide; ADO, adenosine; ENPP1, ectonucleotide pyrophosphatase/phosphodiesterases.

Our knowledge on NAD biology has grown exponentially over the past few years, including biosynthetic and degrading pathways. A general decrease in cellular NAD is described in many age-related diseases, whereas increased NAD levels are associated to inflammatory conditions, including cancer. Figure 1 illustrates the main NAD-biosynthetic and -consuming pathways, as well as the crosstalk between intracellular (i)NAD and eNAD.

### NAD: Energy Cofactor

As energetic co-enzyme, NAD is essential as electron acceptor donor in various metabolic pathways including cytosolic glycolysis, serine biosynthesis, tricarboxylic acid cycle (TCA), oxidative phosphorylation, as well as cell redox state homeostasis redox reactions (30, 31). Cofactor of almost 300 dehydrogenase, NAD is primarily used during glycolysis in the sixth step of the enzymatic chain by glyceraldehyde phosphate dehydrogenase (GAPDH) and at the end of the process by lactate dehydrogenase (LDH), catalyzing the interconversion of pyruvate and lactate and simultaneously of NADH and NAD. The final glycolytic product pyruvate, can be metabolized to produce acetylCoA by the pyruvate dehydrogenase complex (PDC), a reaction accompanied by NAD reduction to NADH (32). During the TCA cycle, NAD is reduced to NADH moieties in several key steps by isocitrate dehydrogenase (IDH), oxoglutarate dehydrogenase (OGD), and malate dehydrogenase (MDH). NADH produced in all these reactions, working as electron equivalent redistributor, is used by the electron transport chain (ETC) to generate ATP (33).

The ratio between NAD/NADH and their relative phosphorylated form (NADP/NADPH), are also critical for enzymatic defense systems against oxidative stress, regulating redox homeostasis through the main cellular scavenging systems which are the glutathione (GSH/GSSG) and the thioredoxin-mediated (Trx-SH/Trx-SS) mechanisms (34–37). In this context, NADPH is the indispensable reducing agent for ROS elimination and redox homeostasis, primarily produced by glucose-6-phosphate dehydrogenase (G6PD) and -phosphogluconate dehydrogenase (6PGD) in the pentose phosphate pathway (36).

### NAD: A Pleiotropic Signaling Molecule

Independently of its redox properties, NAD is also the substrate of enzymes with fundamental roles in gene expression and cell signaling (38). In these reactions, NAD is cleaved at the glycosidic bond between Nam and ADP-ribose acquiring the characteristic of signaling molecule (27).

The large family of NAD consuming enzymes includes mono adenosine diphosphate (ADP)-ribose transferases (ARTs) and poly ADP-ribose polymerases (PARPs), the NAD-dependent deacetylases, sirtuins (SIRT1-7), and the cyclic ADP-ribose hydrolases, NAD glycohydrolases, ectonucleotide pyrophosphatase/phosphodiesterases and ecto-5'-nucleotidase (CD38/CD157 and ENPP1/CD73) (19, 39, 40) (Figure 1).

Through their functional activities of post-translational modifications (ADP-ribosylation and deacetylation), or through the modulation of Ca^2+^ signaling, these enzymes regulate gene transcription, cell differentiation, cell cycle progression, circadian rhythm, DNA repair, chromatin stability, cell adaptation to stress signals, and immune responses (41, 42). Therefore, PARPs and sirtuins represent connecting elements between the metabolic state of a cell and its signaling and transcriptional activities (43).

### Extracellular NAD and Its Biological Role

The eNAD concentration is in the range of 100–500 nM, considerably lower than its intracellular levels (200–500 μM) (39, 44–46). eNAD and iNAD levels are highly linked, due to intra-extra membrane transport of NAD precursors, intermediates of reaction and NAD itself (47). The canonical view is that NAD is unable to cross lipid bilayers, but it enters the cell using dedicated NAD transporters, such as connexin 43 (Cx43) channels, or exits through exocytosis (45, 48–50). In addition, conditions of environmental stress can induce NAD release (51–53). On the other hand, whether there is direct eNAD synthesis remains controversial (39) (Figure 1), despite the presence of extracellular precursors and biosynthetic enzymes. Specifically, it is known that among the different forms of vitamin B3 (NAD precursor), transport of Na is mediated by membrane carrier systems potentially including either a pH-dependent anion antiporter or a proton cotransporter (54, 55). Nam is present extracellularly and its uptake is possible either as direct transport in intact form or converted to salvage pathway metabolites. However, NAMPT's substrates ATP and 5-phosphoribosyl-1-pyrophosphate (PRPP) were shown to be unavailable in sufficient quantities in the extracellular space (56) to support direct eNAD generation.

eNAD can bind different subtypes of purinergic P2 receptors, including P2Y11, leading to the opening of L-type Ca^2+^ channels and activation of a cAMP/cADPR/[Ca^2+^]i signaling cascade, ultimately causing increased proliferation and migration (57). In T cells and monocytes, P2X7 receptor activation generally results in Ca^2+^ internalization, opening a non-selective, large membrane pore, causing cell death (58, 59). eNAD also acts as a neurotransmitter, released by stimulated terminals of mammalian central nervous system and peripheral nervous system neurons, binding to post-synaptic P2Y1 receptors, similar to ATP (60).

The very low levels of eNAD are due to its rapid metabolism/degradation by NAD-catabolic enzymes present on the surface of the cell (61), suggesting that also NAD metabolites may mediate cellular responses in the extracellular environment.

eNAD is degraded by three main classes of specific ectoenzymes: CD38 and CD157 (62, 63), ARTs (64), ENPP1 and CD73 (61, 65, 66). NADase, ENPP1 and CD73 can lead to the formation of adenosine (ADO), a potent immunosuppressant factor, independently of the activity of CD39 (61, 67, 68). Beside generating ADO, eNAD can be degraded to nicotinamide mononucleotide (NMN) by CD38, generating Nam which can cross plasma membranes and be re-converted to NAD through NAMPT and NMN adenylyltransferase (NMNAT) (69). On the other side, NMN can be also used by CD73, which generates nicotinamide riboside (NR) (66, 70), that, likely through equilibrative nucleoside transporters (ENTs), can be imported as NAD precursor (71, 72) (Figure 1). Recently, Slc12a8 was identified as specific NMN transporter (73), suggesting that NMN can be internalized without conversion to NR. Studies on cell type expression pattern of this transporter will clarify this possibility.

## NAD Biosynthesis: the Enzymatic Functions of NAMPT and NAPRT

**Figure 2:**
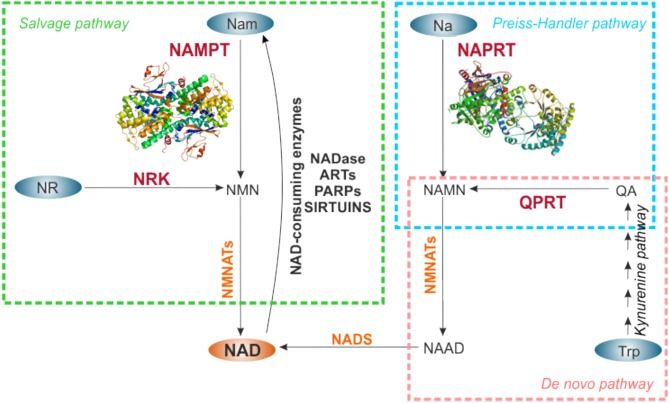
NAD biosynthetic pathways. NAD can be synthetized *de novo* starting from Trp (pink rectangle), or through salvage routes from Nam and NR (green rectangle), or metabolizing Na in the Preiss-Handler pathway (light blue rectangle). NAD-precursors are indicated in the blue ovals. The rate-limiting enzymes of each biosynthetic pathway are indicated in red, the other enzymes involved in the reactions in orange. For NAMPT and NAPRT crystal structures are shown. NAD synthesized from Nam via NAMPT is in turn used by NAD-consuming enzyme activities that release Nam, making it available for continuous NAD regeneration. NAMPT, nicotinamide phosphoribosyltransferase; NAPRT, nicotinate phosphoribosyltransferase; NRK, nicotinamide riboside kinase; QPRT, quinolinate phosphoribosyltransferase; NMNATs, nicotinamide mononucleotide adenylltransferases; NADS, NAD synthase; Nam, nicotinamide; NR, nicotinamide riboside; Na, nicotinic acid; Trp, tryptophan; QA, quinolinic acid; NMN, nicotinamide mononucleotide; NAMN, nicotinate mononucleotide; NAAD, nicotinate adenine dinucleotide; NADase, NAD-glycohydrolase; ARTs, mono adenosine diphosphate (ADP)-ribose transferases; PARPs, poly ADP-ribose polymerases.

NAD turnover within the cell is dynamic, displaying circadian oscillations that are regulated by the core clock machinery CLOCK:BMAL1 (74, 75). Total intracellular levels are maintained between 200 and 500 μM, depending on the cell type or tissue, increasing in response to different stimuli (43). NAD homeostasis is the result of the balance between a number of NAD-consuming reactions and NAD-biosynthetic routes, via three distinct pathways: the *de novo* biosynthetic pathway, the Preiss–Handler pathway, and the salvage pathway, as reviewed in Houtkooper et al. (27), Ruggieri et al. (29), and Audrito et al. (42) and illustrated in Figure 2.

Specifically, *de novo* NAD biosynthesis starts with the catabolism of the amino acid tryptophan to kynurenine by indoleamine-2,3-dioxygenase. Kynurenine is then metabolized through the kynurenine pathway to quinolinic acid (QA), which is converted by quinolate phosphoribosyltransferase (QPRT), rate-limiting enzyme, to Na mononucleotide (NaMN). The Preiss–Handler pathway metabolizes kynurenine pathway–derived NaMN or diet-derived Na, or Na as a product of Nam deamidation by intestinal flora (76) to NAD, via NAPRT rate-limiting activity. In the salvage pathway, NAMPT metabolizes Nam and PRPP to NMN in a rate limiting step, which is then converted into NAD. In a further salvage route, NR, derived from diet, can be used by nicotinamide riboside kinase (NRK), to generate NAD (Figure 2).

Quantitatively, the Nam salvage pathway is the most relevant in mammalian cells. Several lines of evidence support this observation. First, Nam is the most abundant NAD precursor in the bloodstream (39), and can be easily introduced by diet (vitamin B3). Second, Nam is a by-product of all NAD-metabolizing enzymes activity, increasing its availability (77). Third, the rate limiting enzyme NAMPT (EC 2.4.2.12) is expressed in all mammalian tissues (78), as detailed below. Linked to this, *NAMPT* gene deletion in mice is embryonically lethal (79), suggesting the importance of this route to regenerate NAD. In this pathway, Nam N-methyltransefase (NNMT) recently emerged as an evolutionarily conserved regulator of Nam availability. In fact, NNMT N-methylates Nam preventing its accumulation and inhibition of NAD-consuming enzymes, while on the other side, limiting its availability to NAMPT (80, 81).

The functional NAMPT forms a homodimer to catalyze the conversion of Nam and PRPP to NMN. Structural and site-directed mutagenesis studies by Khan et al. demonstrated that Asp219 is fundamental in defining the substrate specificity of NAMPT (82). Wang et al. showed that NAMPT has an autophosphorylation activity and hydrolyzes ATP. Autophosphorylation can increase its enzymatic activity (83). Recently, NAMPT was found to be a direct substrate of SIRT6 deacetylation, a post-translational mechanism that up-regulates its enzymatic activity (84). On the contrary, mutations of His247, a central conserved residue in the active site of the enzyme, significantly decreases or abolishes NAMPT enzymatic activity (83).

NAPRT (EC 2.4.2.11) catalyzes the conversion of Na and PRPP to NaMN and pyrophosphate (PPi). The enzyme, originally named NaMN pyrophosphorylase, was first described by Handler in human erythrocytes, where it increases NAD levels (85).

NAPRT activity is more tissue-specific. Although enzyme activity can be detected in most mouse tissues (86), Na acts as a more efficient precursor than Nam in mice liver, intestine, heart and kidney (87). Furthermore, Na is more efficient than Nam in raising NAD levels in cells exposed to oxidative stress (56, 85, 88).

Contrary to NAMPT, NAPRT is not inhibited by NAD, which explains its significantly higher efficiency in raising NAD levels *in vivo* (56, 89). Moreover, NAPRT is strongly activated by phosphate (85), while ATP behaves as an allosteric modulator of the enzyme (29, 85, 90).

In 2015 Marletta et al. resolved the structure of human (h)NAPRT, highlighting a high degree of structural homology between the human and the bacterial NaPRTases due to evolutionary adaptation (91). As with NAMPT, the functional NAPRT enzyme works as dimers, and despite sharing very limited sequence similarity, hNAPRT shows a molecular fold that closely resembles that firstly described for hNAMPT (83). This opened new hypotheses of shared motifs in NAMPT and NAPRT involved in the binding of extracellular proteins to the receptor, as described in section eNAMPT Functions.

The presence of these multiple NAD biosynthetic routes most likely reflects differences in tissue distribution and/or intracellular compartmentalization of NBEs (39, 46, 76, 92, 93). Our group recently showed that NAMPT and NAPRT are mainly located in cytoplasm and nucleus, while NRK is more expressed in mitochondria, impacting on iNAD levels and response to NAMPT inhibitors (39, 46, 76, 92, 93).

### Identification, Characterization, and Expression of NAMPT and NAPRT

#### NAMPT

The enzyme NAMPT is highly conserved with orthologs in bacteria (94), invertebrate sponges (95), amphibians (96), birds and mammals (97). Not long after its discovery in 1994 by Samal et al. as a pre-B-cell colony enhancing factor (PBEF) secreted by activated lymphocytes and bone marrow stromal cells, Rongvaux et al. (98) showed that murine PBEF could catalyze the conversion of Nam to NMN, a rate-limiting step in NAD biosynthesis. These authors also showed that Actinobacillus pleuropneumonia, a bacterium lacking the NadV gene, transformed with murine PBEF acquires NAD independence, confirming that the enzymatic activity is evolutionarily conserved from bacteria to mammals (98).

In recent years, NAMPT has received increasing attention due to new evidence indicating that it is a pleiotropic protein that may function as NBE, as well as growth factor, cytokine and adipokine [reviewed in (18, 25)]. Although NAMPT lacks the typical signal peptide needed for extracellular secretion, the mature protein can be found in the medium of many cellular cultures due to an active secretion mechanism (99, 100). However, in conditions of cell damage eNAMPT can be released as passive diffusion across cell membranes, as usual for other DAMPS. In addition, the 3' untranslated region (UTR) contains multiple TATT motifs that are characteristic of cytokines (99).

The human *NAMPT* gene spans over 34.7 kb on the long arm of chromosome 7 (7q22) and contains 11 exons and 10 introns (101, 102). Two distinct promoter sites are present in the 5'-flanking region, suggesting the possibility of tissue specific differential expression (101). The region proximal to the promoter is GC-rich and contains 12 binding sites for specificity protein 1 (SP-1), multiple activating protein 2 (AP-2), lymphoid enhancer-binding factor 1 (LF-1), cAMP response element-binding protein (CREB), and signal transducer and activator of transcription (STAT) binding sites (101, 103). Furthermore, the presence of two hypoxia inducible factor (HIF) response elements (HREs) suggest that the gene is upregulated under hypoxic conditions (104). The distal promoter region contains several CAAT boxes and TATA-like sequences, as well as binding sites for nuclear factor 1 (NF-1), nuclear factor kappa-light-chain-enhancer of activated B cells (NF-κB), CCAAT/enhancer binding protein (C/EBPβ), the glucocorticoid receptor (GR), and activating protein 1 (AP-1) (101). The majority of these transcription factors, including NF-1, AP-1, AP-2, NF-κB, and STAT, regulate cytokine expression and their presence in the promoter region of *NAMPT* suggests a role for this enzyme in immunity (42, 105).

Recently, 65 kb downstream of the *NAMPT* transcription start site on chromosome 7 (hg19: 105,856,018–105,860,658), a putative *NAMPT* enhancer was identified as specifically marked by H3K27ac and/or an accessible DNase I hypersensitive (DHS) signal (106). Fine-mapping of the 4.6-kb putative enhancer by stepwise 1-kb deletions or insertions identified the 1-kb enhancer “B” region as responsible for (i) the control of expression of the *NAMPT* gene through c-MYC and MAX activities. (ii) In addition, it is the target of H3K27 acetylation; (iii) it regulates iNAD levels; and (iv) it is required for cell survival in NAMPT-dependent tumors (106). Some genetic polymorphisms were identified in the human *NAMPT* gene, potentially responsible for NAMPT expression. Different representation of these Single Nucleotide Polymorphisms (SNPs) were described in patients with acute respiratory distress syndrome, type 2 diabetes, glucose and lipid metabolism alterations, diastolic blood pressure and hypertensive disorders compared to controls (107).

In the cell, NAMPT is abundant in the cytosol and present in the nucleus (108–110). Recently, Svoboda et al. showed that nuclear NAMPT translocation is a regulated process induced by genotoxic, oxidative, or dicarbonyl stress, mainly to finance NAD production for increased PARP and sirtuin activity (111). Moreover, NAMPT cytosol/nucleus localization changes according to cell cycle phases: it is excluded from the nucleus immediately after mitosis and it migrates back into it as the cell cycle progresses (111). These data were confirmed also by Grolla et al. that demonstrated a transport of NAMPT into the nucleus, GAPDH-mediated, in response to DNA damage (112). On the contrary, the presence of NAMPT in mitochondria remains controversial (30, 46, 109).

Furthermore, an increasing number of cell types have been shown to release eNAMPT, including adipocytes, hepatocytes, cardiomyocytes, activated immune cells and several tumor cells (100, 113–117). While it was shown that a regulated positive secretory process exists (79), the exact mechanisms of release are presently under investigation. The most accredited hypothesis, yet to be confirmed in most cell types, is that eNAMPT is secreted through a “non-classical” secretory pathway, which is not blocked by monensin and brefeldin A, two inhibitors of the classical endoplasmatic reticulum (ER)–Golgi secretory pathway (79, 113, 118, 119). A recent paper showed that eNAMPT is carried in extracellular vesicles (EVs) through systemic circulation in mice and humans. EV-contained-eNAMPT is internalized into cells, enhancing NAD synthesis (120). The same conclusion was obtained by another group identifying that eNAMPT is actively secreted via exosomes from microglia during neuroinflammation due to ischemic injury (121). However, this mechanism of secretion could be context dependent: in fact in 3T3-L1 adipocytes eNAMPT release and secretion do not appear to occur through microvesicles (113).

Whether the extracellular form possesses specific differences in terms of truncations or post-translational modifications is presently unclear. Different groups suggested that deacetylation by sirtuins can impact eNAMPT secretion (84, 122), adding a new layer of complexity.

#### NAPRT

Highly conserved across species, the human *NAPRT* gene is located at chromosome 8q24.3, containing 12 exons. Similar to NAMPT, intracellular NAPRT is located in both the nucleus and the cytoplasm, but not detected in mitochondria (46, 71, 123). Our group firstly reported the presence of an extracellular form of NAPRT in biological fluids in physiological (healthy donor's blood) and inflammatory conditions opening a new field of investigations (124).

Several information about NAPRT expression and regulation emerged in tumors, in relation to the efficacy of NAMPT inhibitors (NAMPTi) as potential anti-cancer agents (125, 126), as described in the dedicated section NAMPT and NAPRT in Tumors.

## Extracellular NAMPT and NAPRT: Adipocytokines and DAMPs

**Figure 3:**
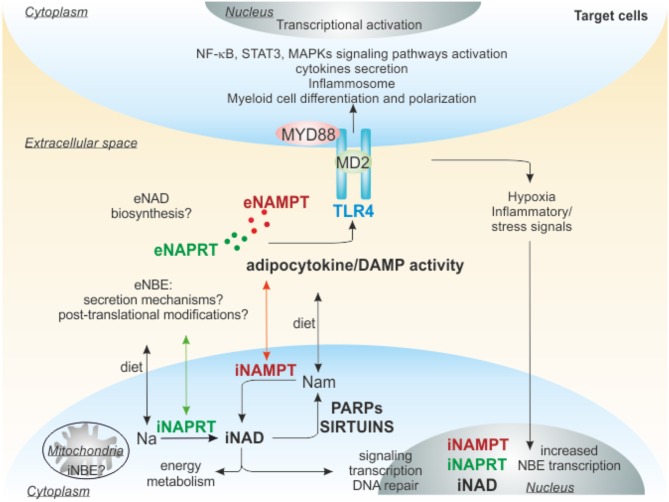
Extracellular functions of NAMPT and NAPRT. iNAMPT and iNAPRT are involved in NAD generation inside of cells, but can be also secreted, through unknown mechanisms, in the extracellular space due to cellular stresses (damage/inflammation/pathological conditions). Extracellularly, they can act as adipocytokine/DAMP binding to TLR4 and triggering intracellular signaling promoting differentiation/polarization of myeloid cells, activation of inflammosome, secretion of pro or anti-inflammatory cytokines. The final outcome depends on the cellular context, for example in tumors eNAMPT creates an immunosuppressive microenvironment, favoring cancer progression, while eNAPRT in sepsis amplifies the inflammatory response. TLR4, Toll-like receptor 4; MD2, myeloid differentiation 2; MYD88, myeloid differentiation primary response gene 88; NAMPT, nicotinamide phosphoribosyltransferase; NAPRT, nicotinate phosphoribosyltransferase; NBEs, NAD-biosynthetic enzymes; Nam, nicotinamide; Na, nicotinic acid; DAMP, damage-associated molecular pattern; PARPs, poly ADP-ribose polymerases.

In addition to a direct effect on NAD and its metabolites, the enzymes involved in synthesis of NAD also have important extracellular functions, as summarized in Figure 3.

### eNAMPT Functions

eNAMPT/PBEF was first identified as an immunomodulatory cytokine able to synergize with interleukin 7 (IL-7) and stem cell factor (SCF) to promote pre-B cell colony formation (99). It is now well-established that eNAMPT is a soluble factor that is up-regulated upon activation in innate and adaptive immune cells, including neutrophils, monocytes, and macrophages, and in epithelial and endothelial cells (18, 127). NAMPT expression can be rapidly induced by inflammatory signals, in particular both pathogen-derived lipopolysaccharide (LPS) and host-derived inflammatory stimuli (TNF-α, IL-1β, IL-6, leptin) in amniotic epithelial cells, macrophages, human osteoarthritic chondrocytes and a synovial fibroblast cell line (101, 103, 128, 129).

eNAMPT has a variety of biological functions (Figure 3): (i) it is an important mediator of inflammatory programs (18, 130) and (ii) it acts as a cytokine that modulates the immune response (42). Notably, the cytokine-like functions appear, at least in part, independent of the protein catalytic activity, as inferred by the use of an enzymatically inactive NAMPT H247E mutant that retains the ability to activate signaling pathways (26, 83, 131, 132). In keeping with this view, NAMPT's substrates PRPP and ATP are apparently unavailable extracellularly to sustain its enzymatic activity (56). Following NAMPT treatment, interleukins IL-1β, IL-6, IL-10, and tumor necrosis factor- α (TNF-α) are up-regulated and secreted by peripheral blood mononuclear cells (PBMCs) and CD14^+^ monocytes (133). Co-stimulatory molecules such as CD40, CD54, and CD80 are also up-regulated in response to eNAMPT exposure, an effect mediated through the activation of PI3-kinase and MAPKs pathways (133). Furthermore, in macrophages NAMPT increases matrix metalloproteinases (MMPs) expression and activity (134). In addition, (iii) eNAMPT has anti-apoptotic effects on immune cells, including neutrophils and macrophages, for example it promotes macrophage survival after induction of endoplasmic reticulum (ER) stress triggering IL-6 secretion and phosphorylation of STAT3 (103). (iv) eNAMPT is also reported as an adipokine, also known as visfatin, playing a critical role in the regulation of glucose-stimulated insulin secretion in pancreatic β cells (21, 135). While a direct role for insulin receptor in eNAMPT-mediated cytokine release was discarded (18, 133), eNAMPT is up-regulated in obese and diabetic patients: it is enriched in visceral fat and secreted by adipocytes (113, 136, 137). The role as adipokine seems more related to the extracellular generation of NMN: in fact systemic administration of NMN to aged mice or mice subjected to a high-fat diet restores normal NAD levels in white adipose tissue and liver, and ameliorates glucose intolerance and type II diabetic syndrome (138). (v) eNAMPT can also act as a pro-angiogenic factor, promoting endothelial cell proliferation, migration, and capillary tube formation in a concentration-dependent manner in human umbilical vein endothelial cells (HUVEC) (139–142). These proliferative effects of eNAMPT seem to be mediated, or at least partially mediated by vascular endothelial cell growth factor (VEGF), the master regulator of endothelial cell program (139). Thus, eNAMPT upregulates VEGF synthesis and secretion, as well as the expression of the VEGF receptor 2, which has been proposed to mediate the angiogenic actions of VEGF (139). Beside VEGF, in endothelial cells eNAMPT upregulates production of other pro-angiogenic soluble factors, such as fibroblast growth factor-2 (FGF-2), monocyte chemoattractant protein-1 (MCP-1) and IL-6 (143, 144). Indeed, both MCP-1 and FGF-2 have also been identified as mediators of eNAMPT-induced angiogenesis (143). Beyond *in vitro* studies, the angiogenic activities of eNAMPT were demonstrated in *ex vivo* and *in vivo* approaches (139, 140).

### eNAPRT Functions

Starting from the structural and functional similarity between human NAMPT and NAPRT (91), our group for the first time investigated whether NAPRT exists in an extracellular form, thus sharing with NAMPT its moonlighting abilities (124). By setting up a new luminex/ELISA assay, we dosed eNAPRT in a cohort of > 100 plasma from normal blood donors (HD), highlighting a mean concentration similar to that recorded for eNAMPT (in the range of 1.5–2.0 ng/ml), with no differences according to gender or age. We used mass spectrometry to confirm the presence of NAPRT peptides in human plasma. Moreover, we demonstrated that endogenous eNAPRT is enzymatically active (86, 124). Analyzing eNAPRT in sera from patients with acute or chronic inflammatory conditions, we demonstrated that this enzyme strongly increased in acute inflammatory diseases such as sepsis and septic shock, driving inflammatory responses related to the activation of macrophages (Figure 3). We also observed that cellular stress [i.e., treatment with TNF-α and cycloheximide to trigger apoptosis, or with ionomycin and carbonyl cyanide 3-chlorophenylhydrazone (CCCP) to trigger necrosis] is accompanied by marked increase of eNAPRT in macrophage culture media (124), as previously described also for HMGB-1 (9) and others DAMPs (145).

### eNAMPT/eNAPRT in Myeloid Cells Function: the Role of TLR4

NAD synthesis has a driving role in myeloid differentiation and in supporting macrophage inflammatory responses (42, 146, 147), prompting investigation on the function of NAMPT and NAPRT in these cells.

Increasing evidence demonstrated a direct role of NAMPT in regulating the differentiation program and the metabolic phenotypes of myeloid cells. Both iNAMPT and eNAMPT influence monocyte/macrophages differentiation, polarization and migration (132, 146, 148). We described a role for eNAMPT in creating an immunosuppressive and tumor-promoting microenvironment in chronic lymphocytic leukemia, where eNAMPT is important for the differentiation of monocytes toward tumor-supporting M2 macrophages (132). Recently, it was demonstrated that iNAMPT acts also on myeloid-derived suppressor cells (MDSCs), where NAMPT blocks *CXCR4* transcription, via a SIRT1/HIF-1α axis. The activation of this circuit, in turn, leads to MDSCs mobilization and enhances the production of nitric oxide, promoting immunosuppression (149).

The NAMPT/NAD/SIRT1 axis seems to play a relevant role in myeloid cell activation. NAMPT-dependent NAD generation is crucial in the metabolic switch characterizing the transition from the early stage of acute inflammation, primarily relies on glycolysis, to the later adaptation phase more dependent on fatty acid oxidation (FAO) for energy production (150–152). Moreover, NAMPT/NAD levels significantly increased during activation of pro-inflammatory M1 macrophages (153). In a further feedback loop some cytokines, including IL-6 and TNF-α, induced during monocyte activation, are able to promote NAMPT expression via HIF-1α. In turn, NAMPT, triggering NF-kB signaling pathway, sustains *IL6* and *TNFA* transcription forcing myeloid cell activation (131). It has been also shown that NAMPT/NAD/SIRT1 axis can regulate neutrophilic granulocyte differentiation via CCAAT/enhancer-binding protein α/β (C/EBPα/β) induction, ultimately, up-regulating granulocyte colony-stimulating factor (G-CSF). In turn, G-CSF further increases NAMPT levels (148). NAMPT inhibition, reducing NAD levels, thereby decreasing SIRT1 activity, leads to the dramatic elevation of acetylated C/EBPα levels and reduces amounts of total C/EBPα protein, accompanied by diminished mRNA expression of C/EBPα target genes (G-CSF, G-CSFR, and ELANE) (148, 154). Moreover, exposure of the acute myeloid leukemia cell line HL-60 to recombinant NAMPT or NAMPT overexpression induced myeloid differentiation of these cells *per se* (154).

A controversial issue in NAMPT biology is whether its cytokine-like properties are all linked to its enzymatic activity or are mediated by the binding to a cell surface receptor. In 2015, Camp et al. showed that eNAMPT produces robust TLR4-mediated NF-kB signaling activation, by directly binding TLR4-MD2 (26) (Figure 3). However, due to possible contamination of LPS, the natural ligand of TLR4, in the recombinant NAMPT preparations used to treat cells *in vitro*, the interpretation of these results remains controversial. Our group recently confirmed the binding of eNAMPT to TLR4 in macrophage cellular model (124), performing surface plasmon resonance (SPR) under the same conditions previously established for the NAMPT-TLR4 interaction (26). More recently, the same group that firstly identified TLR4 as NAMPT receptor published new details about this interaction (155). At the same time, a direct role of NAD in activating the inflammasome was recently reported by Yang et al. The authors demonstrated that NAD manipulation, using NAMPT inhibitors or the treatment with NAD precursors, affects TLR4-mediated NF-κB activation and PYD-domain 3 (NLRP3) inflammasome activity connecting intracellular NAD levels and inflammation (156).

Similar properties were attributed to eNAPRT. By using a surface coated with an anti-NAPRT antibody, we showed that a pre-mixed solution of recombinant (r)NAPRT and rTLR4 resulted in increased binding when compared to rNAPRT alone, indicating that a direct molecular interaction was occurring between the proteins (124). TLR4 triggering by rNAPRT activates an inflammatory signature in human macrophages differentiated from PBMC of healthy donors, as observed also using rNAMPT, promoting robust activation of NF-κB signaling, transcription and secretion of pro-inflammatory cytokines, including IL-1β, IL-8, TNF-α, CCL3, and inflammatory mediators such as caspase-1 (CASP1) and P2X purinoreceptor (124) (Figure 3). These effects are lost in TLR4-silenced macrophages. Accordingly, in macrophages, derived from TLR4^−/−^ mice, rNAPRT exposure was not able to activate NF-κB signaling and cytokine production. Lastly, we demonstrated that the rNAPRT enzymatic deficient mutant is still able to trigger inflammosome in macrophages, indicating that the enzymatic activity is irrelevant to the pro-inflammatory functions of eNAPRT.

rNAPRT, as previously observed for eNAMPT (132, 146, 148), is also able to force monocyte differentiation into macrophages, up-regulating macrophage colony-stimulating factor (M-CFS). This function in triggering macrophage differentiation is a unique feature of eNAMPT/eNAPRT and not shared by LPS, suggesting that even though TLR4 is a receptor for multiple soluble factors and proteins, each specific ligand has a peculiar role. Notably, eNAPRT could be detected in macrophage culture supernatants, suggesting that macrophages are a source of eNAPRT *in vivo*.

Lastly, in this paper, we demonstrated that the signaling functions of hNAMPT and hNAPRT are not an evolutionarily conserved trait. In fact, the bacterial rNAPRT (PncB) or the bacterial rNAMPT (NadV) invariably failed to activate NF-κB signaling in macrophages. Furthermore, a comparison of the surface properties of the bacterial and hNAPRT proteins revealed the presence in hNAPRT of an arginine-rich stretch (_65_**R**FL**R**AF**R**L**R**) forming a large mouth-like positively charged area on the top of the dimer, which is absent in the bacterial ortholog, but is present in a similar form in NAMPT, and could be involved in the binding to TLR4 (124). Even if several issues remain to be investigated, these data support the notion of another NBE acting as extracellular mediator with a direct role in macrophage functions, binding TLR4.

## NAMPT and NAPRT as Biomarkers of Chronic and Acute Inflammatory Diseases

iNAMPT over-expression as well as increased circulating levels of eNAMPT were documented in metabolic/inflammatory conditions including obesity, type 2 diabetes, metabolic syndromes, atherogenic inflammatory diseases, therefore supporting a role for eNAMPT as a potential biomarker of cardio- cerebro-vascular disorders (157–160). Enhanced eNAMPT levels are also described in kidney transplantation recipients (161), polycystic ovary syndrome (162), preeclampsia (163), and acute coronary syndrome (158, 164). Increased eNAMPT levels were additionally reported in non-metabolic chronic inflammatory diseases [i.e., osteoarthritis (103) and acute lung injury (ALI) (165, 166)], characterized by systemic inflammation. eNAMPT also seems to play a role in several types of infections like sepsis (167, 168) or intrauterine infection (chorioamnionitis) (169), and in autoimmune inflammatory diseases including psoriasis (170), rheumatoid arthritis (RA) (171) Crohn's disease (CD) and ulcerative colitis (UC) (172). Table 1 summarized main activities of i/eNAMPT in these pathological conditions.

**Table 1:** NAMPT and NAPRT functions in chronic and acute inflammatory diseases.

**Type of disease**	**Main findings**	**Therapeutic options**	**References**
***e/iNAMPT***			
Type 2 diabetes	-Higher eNAMPT levels in cases than controls -eNAMPT induces a diabetic phenotype in pancreatic islets	Blocking Ab?	(157, 173)
Obesity	-Higher eNAMPT levels in cases than controls -iNAMPT supports adipose plasticity and the pathological progression to obesity.	Blocking Ab? Pharmacological inhibitors?	(157, 174)
Atherogenic inflammatory diseases; cardio- cerebro- vascular disorders; stroke; acute coronary syndrome	-eNAMPT prognostic marker of atherosclerosis, endothelial dysfunction, and vascular damage -Active player promoting vascular inflammation -Deregulated NAD metabolism	Blocking Ab? Pharmacological inhibitors (pre-clinical)	(158–160, 164, 175)
Kidney transplant recipients	-eNAMPT was significantly higher in kidney allograft recipients than in HD -Associated with endothelial damage	Blocking Ab?	(161)
Polycystic ovary syndrome; preeclampsia	-Higher eNAMPT levels in cases than controls - eNAMPT induces the expression of pro- angiogenic factors	Blocking Ab?	(162, 163)
Acute lung injury (ALI); acute respiratory distress syndrome (ARDS)	-Higher eNAMPT levels in cases than controls -eNAMPT induces the secretion of inflammatory cytokines and activation of signaling pathways -iNAMPT supports NAD metabolism, inhibiting apoptosis	Blocking Ab (pre-clinical) Pharmacological inhibitors (pre-clinical)	(165, 166)
Sepsis; septic shock	-Higher eNAMPT levels in cases than controls -eNAMPT induces the secretion of inflammatory cytokines and activation of signaling pathways, supporting inflammation -Diagnostic and prognostic biomarkers (risk factor)	Blocking Ab?	(167, 168)
Intrauterine infection (chorioamnionitis)	-Higher eNAMPT levels in cases than controls	Blocking Ab?	(163, 169)
Psoriasis	-Higher eNAMPT levels in cases than controls -Positive correlation with disease severity	Blocking Ab?	(170)
Rheumatoid arthritis (RA); osteoarthritis	-Higher eNAMPT levels in cases than controls -eNAMPT induces the secretion of inflammatory cytokines and activation of signaling pathways -Block NAMPT have reduced RA progression and inflammatory markers	Blocking Ab? Pharmacological inhibitors (pre-clinical)	(103, 171, 176)
Inflammatory bowel disease (IBD); Crohn's disease (CD); and ulcerative colitis (UC)	-iNAMPT/eNAMPT overexpression/secretion -Association with inflammation, hypoxia (active) and tissue repair (inactive disease)	Blocking Ab? Pharmacological inhibitors (pre-clinical)	(172, 177)
Solid tumors: colorectal, ovarian, breast, gastric, prostate, thyroid, pancreatic cancers, melanoma, gliomas, sarcoma, endometrial =carcinomas, and hematological malignancies	-iNAMPT/eNAMPT over expression -Negative prognostic marker - Regulates metabolic adaptation, DNA repair, gene expression, signaling pathways, cell growth, invasion, stemness, epithelial to mesenchymal transition program, metastatization, angiogenesis, secretion of both pro-inflammatory and immunosuppressive cytokines, resistance to genotoxic stress	Blocking Ab (pre-clinical) Pharmacological inhibitors (pre-clinical and phase I-II-III)	(18, 25, 106, 178, 179)
***e/iNAPRT***			
Sepsis/septic shock	-Higher eNAPRT levels in cases than controls -eNAPRT activates inflammosome -Risk factor for patient survival	Blocking Ab?	(124)
Prostate, ovarian, colorectal, and pancreatic cancers	-*NAPRT* gene amplification -iNAPRT overexpression; -correlation with a BRCAness gene expression signature -NAPRT silencing reduced energy status, protein synthesis, and cell size	Pharmacological inhibitors (pre-clinical)	(88, 106, 123, 180)

*HD, healthy donors; Ab, antibody*.

The first indication that NAPRT can be present in the extracellular space was published by our group in 2019. We dosed eNAPRT in sera from patients with sepsis or septic shock due to bacterial infections. Our results indicated that median eNAPRT levels picked-up to ~25 ng/ml in septic individuals (compared to a median of about 2 ng/ml in HD), underlying high levels of this enzyme in this acute inflammatory condition (124). Circulating NAPRT has a role in mediating endotoxin tolerance at low/physiological doses, in fact the highest plasmatic eNAPRT levels were dosed in patients who died because of septic shock, while those with low concentrations survived. We confirmed a significant association between high levels of eNAPRT and mortality, suggesting that eNAPRT is a novel risk factor in sepsis (124). Even if the biological explanation behind this observation is still partly missing, findings in our work are significant starting points to evaluate the functional role of eNAPRT as DAMP in sepsis, but also in others acute inflammatory conditions (Table 1).

### NAMPT and NAPRT in Tumors

In tumors increased i/eNAMPT have been reported, not only as biomarkers, but also as drivers of tumor progression (18, 25, 178), detailed in Table 1. Cancer cells require high energetic needs to support their proliferation. Increased demand of NAD, obtained through NAMPT overexpression, is needed to finance cellular metabolism and NAD-consuming reactions, including DNA repair activity (41). NAMPT is overexpressed in a broad range of solid tumors including colorectal, ovarian, breast, gastric, prostate, thyroid, pancreatic cancers, melanoma, gliomas, sarcoma, endometrial carcinomas, and hematological malignancies, as reviewed in Dalamaga et al. (178), Yaku et al. (179), Audrito et al. (25), and Chowdhry et al. (106). NAMPT, as intracellular and extracellular factor, exerts a direct role on tumor cells increasing tumor aggressiveness, correlating with worse prognosis and regulating different processes including metabolic adaptation, DNA repair, gene expression, signaling pathways, cell growth, invasion, stemness, epithelial to mesenchymal transition program, metastatization, angiogenesis, secretion of both pro-inflammatory and immunosuppressive cytokines, resistance to genotoxic stress, as reviewed in Dalamaga et al. (178) and Audrito et al. (25). Very recently, Nacarelli et al. described also a role of NAMPT in governing the strength of the proinflammatory senescence-associated secretory (SASP) phenotype observed during senescence, a process implicated in tissue aging and cancer (181).

Recently, amplification of *NAPRT* gene was detected in prostate, ovarian, and pancreatic cancers (106, 123). *NAPRT* gene amplification in tumors correlated with NAPRT expression in matched normal tissues, suggesting a role for tissue context in determining which cancers amplify NAPRT (106). Duarte-Pereira et al. in 2016 extensively studied expression of NAMPT and NAPRT in different tumor types and normal tissues (88). The initial step in that study was to evaluate NAPRT and NAMPT expression in a set of normal human tissues, highlighting a widespread expression for both genes. In tumors, while NAMPT was expressed at mRNA and protein levels in all samples analyzed, NAPRT protein levels were highly diverse, being undetected in several cases. Likewise, NAPRT protein is differentially expressed between cell lines, with markedly decreased expression in carcinoma cell lines MKN28 (gastric), 786-O (renal), HCT116 (colorectal), and in all leukemia cell lines tested (HL-60, NB4, and ML2) (88). Another paper highlighted a role for NAPRT, together with NAMPT, as negative prognostic marker in colorectal cancer, based on TCGA RNA-sequencing data and protein tissue array (180). In a recent work the overexpression of NAPRT in ovarian cancer, correlated with a BRCAness gene expression signature. In this context, NAPRT silencing reduced energy status, protein synthesis, and cell size (123). These results suggest that both transcriptional and post-transcriptional mechanisms regulate the expression of the *NAPRT* gene in cancer types, including mutations in transcription factor binding sites of CREB and Sp1, to promoter methylation and alternative splicing (88). Epigenetic silencing of *NAPRT*, driven by the hypermethylation of CpG islands activity of mutant Protein Phosphatase Mg^2+/^Mn^2+^ Dependent 1D (PPM1D), also known as Wip1, is a recently defined mechanism. As a consequence, PPM1D mutated tumors are particularly sensitive to NAMPTi (182). It was shown that the lack of NAPRT expression in some tumors, such as neuroblastoma, glioblastoma (183) or lymphomas (184), puts NAPRT as a biomarker for the use of Na as a chemoprotectant agent during treatment with NAMPT inhibitors (126). In NAPRT-negative tumors, NAMPT inhibition provides a novel synthetic lethal therapeutic strategy by inducing metabolic stress, while normal cells are rescued by Na via activation of the NAPRT pathway (123, 183–185).

We demonstrated the presence of eNAPRT in sera from patients with a diagnosis of cancer, including solid tumors (prostate, lung and bladder cancer, mesothelioma and metastatic melanoma) and hematological malignancies [myeloma, chronic lymphocytic leukemia (CLL), and diffuse large cell lymphoma (DLCL)] (124) as summarized in Table 1. The median value of circulating eNAPRT is double compared to HD, suggesting a possible role of this enzyme in tumor microenvironment.

Several issues remain to be addressed. First and foremost, it will be important to understand the relationship between eNAPRT and eNAMPT: our findings suggest that they have multiple roles in acute vs. chronic inflammation, engaging TLR4 in different pathological conditions (Table 1) and alerting the immune system to distinct sets of “dangers.”

## Implications for Therapy and Concluding Remarks

NAMPT inhibitors were primarily developed as anticancer agents, depleting NAD and causing metabolic crash and tumor cell death (Table 1).

For iNAMPT selective pharmacological inhibitors exist, the best studied being FK866 (also known as APO866) and GMX1778 (also known as CHS-828), among others (25, 178, 186–189). These inhibitors have been studied in cancer cell lines and animal models showing cytotoxicity and tumor regression (178, 190). Despite these important results *in vitro* and *in vivo*, phase I clinical trials in advanced solid tumors and leukemia showed no objective tumor remission and toxicity (191, 192). One of the known mechanism leading to the partial failure of NAMPTi treatment is due to the concomitant expression of NAPRT (88, 106, 123, 125, 126, 184), that can overcome NAMPT inhibition. A complete analysis of expression of these two NBE in tumors should be made to design better therapeutic strategies that deplete NAD improving efficacy. Development of novel NAPRTi, to obtain complete depletion of NAD in tumor insensitive to NAMPTi due to the overexpression of NAPRT should also be considered. Previous studies indicated the ability of structural analogs of Na to inhibit NAPRT enzymatic activity (85, 89). Among this class of compound, 2-hydroxinicotinic acid (2-HNA) is the most promising, showing significant inhibition of NAPRT enzymatic activity and function in ovarian cancer *in vitro* and in xenograft models (123). The use of NAPRT inhibitors appears as a promising strategy to overcome NAPRT-mediated resistance to NAMPT inhibitors in patients (Table 1).

Blocking the extracellular cytokine-like function of eNAMPT and eNAPRT would be very useful to restore immune competence in cancer, as well as, infection setting (Table 1). In the tumor microenvironment, neutralization of eNAMPT using blocking antibodies could be effective to repolarize myeloid cells (TAMs/MDSCs) against tumor. Some groups/companies are working on the production of these antibodies (193), hypothesizing a combination strategy with immunotherapy, or a double inhibition of i/eNAMPT. Blocking eNAPRT in acute inflammatory conditions, such as in septic patients, could be an important strategy to prevent the damaging action of a massive secretion of eNAPRT leading to decreased survival of patients, but this remains, at this moment, only a speculative hypothesis.

In conclusion, in this review we summarized current knowledge on these two old enzymes involved in NAD biosynthesis that can powerfully modulate immune responses. If NAMPT has now an acknowledged role in regulating several cellular processes in physiological and pathological conditions, and as biomarker in several diseases, the biology of NAPRT, especially as new soluble factor, acting as DAMP in acute inflammation, needs to be extensively studied to determine potential pharmacological settings.

## Author Contributions

VA and SD have made a substantial, direct and intellectual contribution to the work, contributed equally to writing the manuscript, and approved it for publication. VM contributed to writing the final version of the manuscript and approved it for publication.

### Conflict of Interest

The authors declare that the research was conducted in the absence of any commercial or financial relationships that could be construed as a potential conflict of interest.

## References

[1] Broz P Monack DM. Newly described pattern recognition receptors team up against intracellular pathogens. Nat Rev Immunol. (2013) 13:551–65. 10.1038/nri3479 23846113

[2] Cao X. Self-regulation and cross-regulation of pattern-recognition receptor signalling in health and disease. Nat Rev Immunol. (2016) 16:35–50. 10.1038/nri.2015.8 26711677

[3] Takeuchi O Akira S. Pattern recognition receptors and inflammation. Cell. (2010) 140:805–20. 10.1016/j.cell.2010.01.022 20303872

[4] Gong T Liu L Jiang W Zhou R. DAMP-sensing receptors in sterile inflammation and inflammatory diseases. Nat Rev Immunol. (2019) 20:95–112. 10.1038/s41577-019-0215-7 31558839

[5] Shi Y Evans JE Rock KL. Molecular identification of a danger signal that alerts the immune system to dying cells. Nature. (2003) 425:516–21. 10.1038/nature01991 14520412

[6] Bianchi ME. DAMPs, PAMPs and alarmins: all we need to know about danger. J Leukoc Biol. (2007) 81:1–5. 10.1189/jlb.0306164 17032697

[7] Chen GY Nunez G. Sterile inflammation: sensing and reacting to damage. Nat Rev Immunol. (2010) 10:826–37. 10.1038/nri2873 21088683 PMC3114424

[8] Pradeu T Cooper EL. The danger theory: 20 years later. Front Immunol. (2012) 3:287. 10.3389/fimmu.2012.00287 23060876 PMC3443751

[9] Scaffidi P Misteli T Bianchi ME. Release of chromatin protein HMGB1 by necrotic cells triggers inflammation. Nature. (2002) 418:191–195. 10.1038/nature00858 12110890

[10] Bianchi ME Crippa MP Manfredi AA Mezzapelle R Rovere Querini P Venereau E. High-mobility group box 1 protein orchestrates responses to tissue damage via inflammation, innate and adaptive immunity, and tissue repair. Immunol Rev. (2017) 280:74–82. 10.1111/imr.12601 29027228

[11] Yang H Ochani M Li J Qiang X Tanovic M Harris HE. Reversing established sepsis with antagonists of endogenous high-mobility group box 1. Proc Natl Acad Sci USA. (2004) 101:296–301. 10.1073/pnas.2434651100 14695889 PMC314179

[12] Andersson U Tracey KJ. HMGB1 is a therapeutic target for sterile inflammation and infection. Annu Rev Immunol. (2011) 29:139–62. 10.1146/annurev-immunol-030409-101323 21219181 PMC4536551

[13] Wang H Bloom O Zhang M Vishnubhakat JM Ombrellino M Che J. HMG-1 as a late mediator of endotoxin lethality in mice. Science. (1999) 285:248–51. 10.1126/science.285.5425.248 10398600

[14] Yatim N Cullen S Albert ML. Dying cells actively regulate adaptive immune responses. Nat Rev Immunol. (2017) 17:262–75. 10.1038/nri.2017.9 28287107

[15] Corriden R Insel PA. Basal release of ATP: an autocrine-paracrine mechanism for cell regulation. Sci Signal. (2010) 3:re1. 10.1126/scisignal.3104re1 20068232 PMC3085344

[16] Junger WG. Immune cell regulation by autocrine purinergic signalling. Nat Rev Immunol. (2011) 11:201–12. 10.1038/nri2938 21331080 PMC4209705

[17] Di Virgilio F Sarti AC Falzoni S De Marchi E Adinolfi E. Extracellular ATP and P2 purinergic signalling in the tumour microenvironment. Nat Rev Cancer. (2018) 18:601–18. 10.1038/s41568-018-0037-0 30006588

[18] Garten A Schuster S Penke M Gorski T de Giorgis T Kiess W. Physiological and pathophysiological roles of NAMPT and NAD metabolism. Nat Rev Endocrinol. (2015) 11:535–46. 10.1038/nrendo.2015.117 26215259

[19] Verdin E. NAD(+) in aging, metabolism, and neurodegeneration. Science. (2015) 350:1208–13. 10.1126/science.aac4854 26785480

[20] Tanaka T Nabeshima Y. Nampt/PBEF/Visfatin: a new player in beta cell physiology and in metabolic diseases? Cell Metab. (2007) 6:341–3. 10.1016/j.cmet.2007.10.004 17983577

[21] Imai S. Nicotinamide phosphoribosyltransferase (Nampt): a link between NAD biology, metabolism, and diseases. Curr Pharm Des. (2009) 15:20–8. 10.2174/138161209787185814 19149599 PMC2734389

[22] Dahl TB Holm S Aukrust P Halvorsen B. Visfatin/NAMPT: a multifaceted molecule with diverse roles in physiology and pathophysiology. Annu Rev Nutr. (2012) 32:229–43. 10.1146/annurev-nutr-071811-150746 22462624

[23] Jieyu H Chao T Mengjun L Shalong W Xiaomei G Jianfeng L. Nampt/Visfatin/PBEF: a functionally multi-faceted protein with a pivotal role in malignant tumors. Curr Pharm Des. (2012) 18:6123–32. 10.2174/138161212803582531 22934941

[24] Carbone F Liberale L Bonaventura A Vecchie A Casula M Cea M. Regulation and function of extracellular nicotinamide phosphoribosyltransferase/visfatin. Compr Physiol. (2017) 7:603–21. 10.1002/cphy.c160029 28333382

[25] Audrito V Manago A Gaudino F Deaglio S. Targeting metabolic reprogramming in metastatic melanoma: the key role of nicotinamide phosphoribosyltransferase (NAMPT). Semin Cell Dev Biol. (2019) 98:192–201. 10.1016/j.semcdb.2019.05.001 31059816

[26] Camp SM Ceco E Evenoski CL Danilov SM Zhou T Chiang ET. Unique toll-like receptor 4 activation by NAMPT/PBEF induces NFkappaB signaling and inflammatory lung injury. Sci Rep. (2015) 5:13135. 10.1038/srep13135 26272519 PMC4536637

[27] Houtkooper RH Canto C Wanders RJ Auwerx J. The secret life of NAD+: an old metabolite controlling new metabolic signaling pathways. Endocr Rev. (2010) 31:194–223. 10.1210/er.2009-0026 20007326 PMC2852209

[28] Dolle C Skoge RH Vanlinden MR Ziegler M. NAD biosynthesis in humans–enzymes, metabolites and therapeutic aspects. Curr Top Med Chem. (2013) 13:2907–17. 10.2174/15680266113136660206 24171775

[29] Ruggieri S Orsomando G Sorci L Raffaelli N. Regulation of NAD biosynthetic enzymes modulates NAD-sensing processes to shape mammalian cell physiology under varying biological cues. Biochim Biophys Acta. (2015) 1854:1138–49. 10.1016/j.bbapap.2015.02.021 25770681

[30] Yang H Yang T Baur JA Perez E Matsui T Carmona JJ. Nutrient-sensitive mitochondrial NAD+ levels dictate cell survival. Cell. (2007) 130:1095–107. 10.1016/j.cell.2007.07.035 17889652 PMC3366687

[31] Yaku K Okabe K Nakagawa T. NAD metabolism: implications in aging and longevity. Ageing Res Rev. (2018) 47:1–7. 10.1016/j.arr.2018.05.006 29883761

[32] Allison SJ Knight JR Granchi C Rani R Minutolo F Milner J. Identification of LDH-A as a therapeutic target for cancer cell killing via (i) p53/NAD(H)-dependent and (ii) p53-independent pathways. Oncogenesis. (2014) 3:e102. 10.1038/oncsis.2014.16 24819061 PMC4035693

[33] Yang Y Sauve AA. NAD(+) metabolism: bioenergetics, signaling and manipulation for therapy. Biochim Biophys Acta. (2016) 1864:1787–800. 10.1016/j.bbapap.2016.06.014 27374990 PMC5521000

[34] Green RM Graham M O'Donovan MR Chipman JK Hodges NJ. Subcellular compartmentalization of glutathione: correlations with parameters of oxidative stress related to genotoxicity. Mutagenesis. (2006) 21:383–90. 10.1093/mutage/gel043 17012304

[35] Morgan B Ezerina D Amoako TN Riemer J Seedorf M Dick TP. Multiple glutathione disulfide removal pathways mediate cytosolic redox homeostasis. Nat Chem Biol. (2013) 9:119–25. 10.1038/nchembio.1142 23242256

[36] Xiao W Wang RS Handy DE Loscalzo J. NAD(H) and NADP(H) redox couples and cellular energy metabolism. Antioxid Redox Signal. (2018) 28:251–72. 10.1089/ars.2017.7216 28648096 PMC5737637

[37] Zhao Y Zhang Z Zou Y Yang Y. Visualization of nicotine adenine dinucleotide redox homeostasis with genetically encoded fluorescent sensors. Antioxid Redox Signal. (2018) 28:213–29. 10.1089/ars.2017.7226 28648094

[38] Berger F Ramirez-Hernandez MH Ziegler M. The new life of a centenarian: signalling functions of NAD(P). Trends Biochem Sci. (2004) 29:111–8. 10.1016/j.tibs.2004.01.007 15003268

[39] Di Stefano M Conforti L. Diversification of NAD biological role: the importance of location. FEBS J. (2013) 280:4711–28. 10.1111/febs.12433 23848828

[40] Hassinen IE. Signaling and regulation through the NAD(+) and NADP(+) networks. Antioxid Redox Signal. (2019) 30:857–74. 10.1089/ars.2017.7479 29284289

[41] Chiarugi A Dolle C Felici R Ziegler M. The NAD metabolome–a key determinant of cancer cell biology. Nat Rev Cancer. (2012) 12:741–52. 10.1038/nrc3340 23018234

[42] Audrito V Manago A Gaudino F Sorci L Messana VG Raffaelli N. NAD-biosynthetic and consuming enzymes as central players of metabolic regulation of innate and adaptive immune responses in cancer. Front Immunol. (2019) 10:1720. 10.3389/fimmu.2019.01720 31402913 PMC6671870

[43] Canto C Menzies KJ Auwerx J. NAD(+) metabolism and the control of energy homeostasis: a balancing act between mitochondria and the nucleus. Cell Metab. (2015) 22:31–53. 10.1016/j.cmet.2015.05.023 26118927 PMC4487780

[44] O'Reilly T Niven DF. Levels of nicotinamide adenine dinucleotide in extracellular body fluids of pigs may be growth-limiting for *Actinobacillus pleuropneumoniae* and *Haemophilus parasuis*. Can J Vet Res. (2003) 67:229–31. 12889731 PMC227058

[45] Billington RA Bruzzone S De Flora A Genazzani AA Koch-Nolte F Ziegler M. Emerging functions of extracellular pyridine nucleotides. Mol Med. (2006) 12:324–7. 10.2119/2006-00075.Billington 17380199 PMC1829198

[46] Gaudino F Manfredonia I Manago A Audrito V Raffaelli N Vaisitti T. Subcellular characterization of nicotinamide adenine dinucleotide biosynthesis in metastatic melanoma by using organelle-specific biosensors. Antioxid Redox Signal. (2019) 31:1150–65. 10.1089/ars.2019.7799 31456414

[47] Kulkarni CA Brookes P Cellular compartmentation and the redox/non-redox functions of NAD. Antioxid Redox Signal. (2019) 31:623–42. 10.1089/ars.2018.7722 30784294 PMC6657305

[48] Bruzzone S Guida L Zocchi E Franco L De Flora A. Connexin 43 hemi channels mediate Ca^2+^-regulated transmembrane NAD+ fluxes in intact cells. FASEB J. (2001) 15:10–12. 10.1096/fj.00-0566fje 11099492

[49] Hwang SJ Durnin L Dwyer L Rhee PL Ward SM Koh SD. beta-nicotinamide adenine dinucleotide is an enteric inhibitory neurotransmitter in human and nonhuman primate colons. Gastroenterology. (2011) 140:608–17 e606. 10.1053/j.gastro.2010.09.039 20875415 PMC3031738

[50] Mottahedeh J Haffner MC Grogan TR Hashimoto T Crowell PD Beltran H. CD38 is methylated in prostate cancer and regulates extracellular NAD(). Cancer Metab. (2018) 6:13. 10.1186/s40170-018-0186-3 30258629 PMC6150989

[51] Adriouch S Hubert S Pechberty S Koch-Nolte F Haag F Seman M. NAD(+) released during inflammation participates in T cell homeostasis by inducing ART2-mediated death of naive T cells *in vivo*. J Immunol. (2007) 179:186–94. 10.4049/jimmunol.179.1.186 17579037

[52] Grahnert A Klein C Schilling E Wehrhahn J Hauschildt S. Review: NAD +: A modulator of immune functions. Innate Immun. (2011) 17:212–33. 10.1177/1753425910361989 20388721

[53] Adriouch S Haag F Boyer O Seman M Koch-Nolte F. Extracellular NAD(+): a danger signal hindering regulatory T cells. Microbes Infect. (2012) 14:1284–92. 10.1016/j.micinf.2012.05.011 22634347

[54] Takanaga H Maeda H Yabuuchi H Tamai I Higashida H Tsuji A. Nicotinic acid transport mediated by pH-dependent anion antiporter and proton cotransporter in rabbit intestinal brush-border membrane. J Pharm Pharmacol. (1996) 48:1073–7. 10.1111/j.2042-7158.1996.tb05902.x 8953511

[55] Said HM Nabokina SM Balamurugan K Mohammed ZM Urbina C Kashyap ML. Mechanism of nicotinic acid transport in human liver cells: experiments with HepG2 cells and primary hepatocytes. Am J Physiol Cell Physiol. (2007) 293:C1773–8. 10.1152/ajpcell.00409.2007 17928533

[56] Hara N Yamada K Shibata T Osago H Tsuchiya M Nicotinamide phosphoribosyltransferase/visfatin does not catalyze nicotinamide mononucleotide formation in blood plasma. PLoS ONE. (2011) 6:e22781 10.1371/journal.pone.0022781 21826208 PMC3149623

[57] Fruscione F Scarfi S Ferraris C Bruzzone S Benvenuto F Guida L. Regulation of human mesenchymal stem cell functions by an autocrine loop involving NAD+ release and P2Y11-mediated signaling. Stem Cells Dev. (2011) 20:1183–98. 10.1089/scd.2010.0295 20964598

[58] Gerth A Nieber K Oppenheimer NJ Hauschildt S. Extracellular NAD+ regulates intracellular free calcium concentration in human monocytes. Biochem J. (2004) 382(Pt 3):849–56. 10.1042/BJ20040979 15233622 PMC1133960

[59] Aswad F Kawamura H Dennert G. High sensitivity of CD4+CD25+ regulatory T cells to extracellular metabolites nicotinamide adenine dinucleotide and ATP: a role for P2X7 receptors. J Immunol. (2005) 175:3075–83. 10.4049/jimmunol.175.5.3075 16116196

[60] Durnin L Hwang SJ Ward SM Sanders KM Mutafova-Yambolieva VN. Adenosine 5-diphosphate-ribose is a neural regulator in primate and murine large intestine along with beta-NAD(^+^). J Physiol. (2012) 590(Pt 8):1921–41. 10.1113/jphysiol.2011.222414 22351627 PMC3573313

[61] Deaglio S Robson SC. Ectonucleotidases as regulators of purinergic signaling in thrombosis, inflammation, and immunity. Adv Pharmacol. (2011) 61:301–32. 10.1016/B978-0-12-385526-8.00010-2 21586363 PMC5879773

[62] Deaglio S Malavasi F. The CD38/CD157 mammalian gene family: an evolutionary paradigm for other leukocyte surface enzymes. Purinerg Signal. (2006) 2:431–41. 10.1007/s11302-006-9002-6 18404481 PMC2096639

[63] Chini EN. CD38 as a regulator of cellular NAD: a novel potential pharmacological target for metabolic conditions. Curr Pharm Des. (2009) 15:57–63. 10.2174/138161209787185788 19149603 PMC2883294

[64] Seman M Adriouch S Haag F Koch-Nolte F. Ecto-ADP-ribosyltransferases (ARTs): emerging actors in cell communication and signaling. Curr Med Chem. (2004) 11:857–72. 10.2174/0929867043455611 15078170

[65] Katada T Kontani K Wada T Hosoda N Hoshino S Nishina H. Enzymic and signal transduction properties of CD38/NADase and PC-1/phosphodiesterase. Chem Immunol. (2000) 75:60–78. 10.1159/000058762 10851779

[66] Garavaglia S Bruzzone S Cassani C Canella L Allegrone G Sturla L. The high-resolution crystal structure of periplasmic Haemophilus influenzae NAD nucleotidase reveals a novel enzymatic function of human CD73 related to NAD metabolism. Biochem J. (2012) 441:131–41. 10.1042/BJ20111263 21933152

[67] Horenstein AL Chillemi A Zaccarello G Bruzzone S Quarona V Zito A. A CD38/CD203a/CD73 ectoenzymatic pathway independent of CD39 drives a novel adenosinergic loop in human T lymphocytes. Oncoimmunology. (2013) 2:e26246. 10.4161/onci.26246 24319640 PMC3850273

[68] Vaisitti T Arruga F Guerra G Deaglio S. Ectonucleotidases in blood malignancies: a tale of surface markers and therapeutic targets. Front Immunol. (2019) 10:2301. 10.3389/fimmu.2019.02301 31636635 PMC6788384

[69] Camacho-Pereira J Tarrago MG Chini C.C. S. Nin V Escande C Warner GM. CD38 dictates age-related NAD decline and mitochondrial dysfunction through an SIRT3-dependent mechanism. Cell Metab. (2016) 23:1127–39. 10.1016/j.cmet.2016.05.006 27304511 PMC4911708

[70] Grozio A Sociali G Sturla L Caffa I Soncini D Salis A. CD73 protein as a source of extracellular precursors for sustained NAD+ biosynthesis in FK866-treated tumor cells. J Biol Chem. (2013) 288:25938–49. 10.1074/jbc.M113.470435 23880765 PMC3764798

[71] Nikiforov A Dolle C Niere M Ziegler M. Pathways and subcellular compartmentation of NAD biosynthesis in human cells: from entry of extracellular precursors to mitochondrial NAD generation. J Biol Chem. (2011) 286:21767–78. 10.1074/jbc.M110.213298 21504897 PMC3122232

[72] Ratajczak J Joffraud M Trammell SA Ras R Canela N Boutant M. NRK1 controls nicotinamide mononucleotide and nicotinamide riboside metabolism in mammalian cells. Nat Commun. (2016) 7:13103. 10.1038/ncomms13103 27725675 PMC5476803

[73] Grozio A Mills KF Yoshino J Bruzzone S Sociali G Tokizane K. Slc12a8 is a nicotinamide mononucleotide transporter. Nat Metab. (2019) 1:47–57. 10.1038/s42255-018-0009-4 31131364 PMC6530925

[74] Nakahata Y Sahar S Astarita G Kaluzova M Sassone-Corsi P. Circadian control of the NAD+ salvage pathway by CLOCK-SIRT1. Science. (2009) 324:654–7. 10.1126/science.1170803 19286518 PMC6501775

[75] Ramsey KM Yoshino J Brace CS Abrassart D Kobayashi Y Marcheva B. Circadian clock feedback cycle through NAMPT-mediated NAD+ biosynthesis. Science. (2009) 324:651–4. 10.1126/science.1171641 19299583 PMC2738420

[76] Bogan KL Brenner C. Nicotinic acid, nicotinamide, and nicotinamide riboside: a molecular evaluation of NAD+ precursor vitamins in human nutrition. Annu Rev Nutr. (2008) 28:115–30. 10.1146/annurev.nutr.28.061807.155443 18429699

[77] Sauve AA. NAD+ and vitamin B3: from metabolism to therapies. J Pharmacol Exp Ther. (2008) 324:883–93. 10.1124/jpet.107.120758 18165311

[78] Rongvaux A Andris F Van Gool F Leo O. Reconstructing eukaryotic NAD metabolism. Bioessays. (2003) 25:683–90. 10.1002/bies.10297 12815723

[79] Revollo JR Korner A Mills KF Satoh A Wang T Garten A. Nampt/PBEF/Visfatin regulates insulin secretion in beta cells as a systemic NAD biosynthetic enzyme. Cell Metab. (2007) 6:363–75. 10.1016/j.cmet.2007.09.003 17983582 PMC2098698

[80] Pissios P. Nicotinamide N-methyltransferase: more than a vitamin B3 clearance enzyme. Trends Endocrinol Metab. (2017) 28:340–53. 10.1016/j.tem.2017.02.004 28291578 PMC5446048

[81] Bockwoldt M Houry D Niere M Gossmann TI Reinartz I Schug A. Identification of evolutionary and kinetic drivers of NAD-dependent signaling. Proc Natl Acad Sci USA. (2019) 116:15957–66. 10.1073/pnas.1902346116 31341085 PMC6689970

[82] Khan JA Tao X Tong L. Molecular basis for the inhibition of human NMPRTase, a novel target for anticancer agents. Nat Struct Mol Biol. (2006) 13:582–8. 10.1038/nsmb1105 16783377

[83] Wang T Zhang X Bheda P Revollo JR Imai S Wolberger C. Structure of Nampt/PBEF/visfatin, a mammalian NAD+ biosynthetic enzyme. Nat Struct Mol Biol. (2006) 13:661–2. 10.1038/nsmb1114 16783373

[84] Sociali G Grozio A Caffa I Schuster S Becherini P Damonte P. SIRT6 deacetylase activity regulates NAMPT activity and NAD(P)(H) pools in cancer cells. FASEB J. (2019) 33:3704–17. 10.1096/fj.201800321R 30514106 PMC6988859

[85] Galassi L Di Stefano M Brunetti L Orsomando G Amici A Ruggieri S. Characterization of human nicotinate phosphoribosyltransferase: kinetic studies, structure prediction and functional analysis by site-directed mutagenesis. Biochimie. (2012) 94:300–9. 10.1016/j.biochi.2011.06.033 21742010

[86] Zamporlini F Ruggieri S Mazzola F Amici A Orsomando G Raffaelli N. Novel assay for simultaneous measurement of pyridine mononucleotides synthesizing activities allows dissection of the NAD biosynthetic machinery in mammalian cells. FEBS J. (2014) 281:5104–19. 10.1111/febs.13050 25223558

[87] Collins PB Chaykin S. The management of nicotinamide and nicotinic acid in the mouse. J Biol Chem. (1972) 247:778–83. 4333514

[88] Duarte-Pereira S Pereira-Castro I Silva SS Correia MG Neto C da Costa LT. Extensive regulation of nicotinate phosphoribosyltransferase (NAPRT) expression in human tissues and tumors. Oncotarget. (2016) 7:1973–83. 10.18632/oncotarget.6538 26675378 PMC4811510

[89] Gaut ZN Solomon HM. Inhibition of nicotinate phosphoribosyltransferase in human platelet lysate by nicotinic acid analogs. Biochem Pharmacol. (1971) 20:2903–6. 10.1016/0006-2952(71)90202-4 4255930

[90] Smith LD Gholson RK. Allosteric properties of bovine liver nicotinate phosphoribosyltransferase. J Biol Chem. (1969) 244:68–71. 5773290

[91] Marletta AS Massarotti A Orsomando G Magni G Rizzi M Garavaglia S. Crystal structure of human nicotinic acid phosphoribosyltransferase. FEBS Open Biol. (2015) 5:419–28. 10.1016/j.fob.2015.05.002 26042198 PMC4442680

[92] Berger F Lau C Dahlmann M Ziegler M. Subcellular compartmentation and differential catalytic properties of the three human nicotinamide mononucleotide adenylyltransferase isoforms. J Biol Chem. (2005) 280:36334–41. 10.1074/jbc.M508660200 16118205

[93] Stein LR Imai S. The dynamic regulation of NAD metabolism in mitochondria. Trends Endocrinol Metab. (2012) 23:420–8. 10.1016/j.tem.2012.06.005 22819213 PMC3683958

[94] Martin PR Shea RJ Mulks MH. Identification of a plasmid-encoded gene from *Haemophilus ducreyi* which confers NAD independence. J Bacteriol. (2001) 183:1168–74. 10.1128/JB.183.4.1168-1174.2001 11157928 PMC94989

[95] Muller WE Perovic S Wilkesman J Kruse M Muller IM Batel R. Increased gene expression of a cytokine-related molecule and profilin after activation of Suberites domuncula cells with xenogeneic sponge molecule(s). DNA Cell Biol. (1999) 18:885–93. 10.1089/104454999314746 10619600

[96] Fujiki K Shin DH Nakao M Yano T. Molecular cloning and expression analysis of the putative carp (Cyprinus carpio) pre-B cell enhancing factor. Fish Shellfish Immunol. (2000) 10:383–5. 10.1006/fsim.2000.0263 10938748

[97] McGlothlin JR Gao L Lavoie T Simon BA Easley RB Ma SF. Molecular cloning and characterization of canine pre-B-cell colony-enhancing factor. Biochem Genet. (2005) 43:127–41. 10.1007/s10528-005-1505-2 15934174

[98] Rongvaux A Shea RJ Mulks MH Gigot D Urbain J Leo O. Pre-B-cell colony-enhancing factor, whose expression is up-regulated in activated lymphocytes, is a nicotinamide phosphoribosyltransferase, a cytosolic enzyme involved in NAD biosynthesis. Eur J Immunol. (2002) 32:3225–34. 10.1002/1521-4141(200211)32:11<3225::AID-IMMU3225>3.0.CO;2-L 12555668

[99] Samal B Sun Y Stearns G Xie C Suggs S McNiece I. Cloning and characterization of the cDNA encoding a novel human pre-B-cell colony-enhancing factor. Mol Cell Biol. (1994) 14:1431–7. 10.1128/MCB.14.2.1431 8289818 PMC358498

[100] Grolla AA Travelli C Genazzani AA Sethi JK. Extracellular nicotinamide phosphoribosyltransferase, a new cancer metabokine. Br J Pharmacol. (2016) 173:2182–94. 10.1111/bph.13505 27128025 PMC4919578

[101] Ognjanovic S Bao S Yamamoto SY Garibay-Tupas J Samal B Bryant-Greenwood GD. Genomic organization of the gene coding for human pre-B-cell colony enhancing factor and expression in human fetal membranes. J Mol Endocrinol. (2001) 26:107–17. 10.1677/jme.0.0260107 11241162

[102] Zhang LQ Heruth DP Ye SQ. Nicotinamide phosphoribosyltransferase in human diseases. J Bioanal Biomed. (2011) 3:13–25. 10.4172/1948-593X.1000038 22140607 PMC3227030

[103] Nowell MA Richards PJ Fielding CA Ognjanovic S Topley N Williams AS. Regulation of pre-B cell colony-enhancing factor by STAT-3-dependent interleukin-6 trans-signaling: implications in the pathogenesis of rheumatoid arthritis. Arthritis Rheum. (2006) 54:2084–95. 10.1002/art.21942 16802343

[104] Bae SK Kim SR Kim JG Kim JY Koo TH Jang HO. Hypoxic induction of human visfatin gene is directly mediated by hypoxia-inducible factor-1. FEBS Lett. (2006) 580:4105–13. 10.1016/j.febslet.2006.06.052 16828081

[105] Luk T Malam Z Marshall JC. Pre-B cell colony-enhancing factor (PBEF)/visfatin: a novel mediator of innate immunity. J Leukoc Biol. (2008) 83:804–16. 10.1189/jlb.0807581 18252866

[106] Chowdhry S Zanca C Rajkumar U Koga T Diao Y Raviram R. NAD metabolic dependency in cancer is shaped by gene amplification and enhancer remodelling. Nature. (2019) 569:570–5. 10.1038/s41586-019-1150-2 31019297 PMC7138021

[107] Duarte-Pereira S Silva SS Azevedo L Castro L Amorim A Silva RM. NAMPT and NAPRT1: novel polymorphisms and distribution of variants between normal tissues and tumor samples. Sci Rep. (2014) 4:6311. 10.1038/srep06311 25201160 PMC4158320

[108] Kitani T Okuno S Fujisawa H. Growth phase-dependent changes in the subcellular localization of pre-B-cell colony-enhancing factor. FEBS Lett. (2003) 544:74–78. 10.1016/S0014-5793(03)00476-9 12782293

[109] Pittelli M Formentini L Faraco G Lapucci A Rapizzi E Cialdai F. Inhibition of nicotinamide phosphoribosyltransferase: cellular bioenergetics reveals a mitochondrial insensitive NAD pool. J Biol Chem. (2010) 285:34106–114. 10.1074/jbc.M110.136739 20724478 PMC2962509

[110] Zhu Y Liu J Park J Rai P Zhai RG. Subcellular compartmentalization of NAD(+) and its role in cancer: A sereNADe of metabolic melodies. Pharmacol Ther. (2019) 200:27–41. 10.1016/j.pharmthera.2019.04.002 30974124 PMC7010080

[111] Svoboda P Krizova E Sestakova S Vapenkova K Knejzlik Z Rimpelova S. Nuclear transport of nicotinamide phosphoribosyltransferase is cell cycle-dependent in mammalian cells, and its inhibition slows cell growth. J Biol Chem. (2019) 294:8676–89. 10.1074/jbc.RA118.003505 30975903 PMC6552417

[112] Grolla AA Miggiano R Di Marino D Bianchi M Gori A Orsomando G. A nicotinamide phosphoribosyltransferase-GAPDH interaction sustains the stress-induced NMN/NAD(+) salvage pathway in the nucleus. J Biol Chem. (2020) 295:3635–51. 10.1074/jbc.RA119.010571 31988240 PMC7076215

[113] Tanaka M Nozaki M Fukuhara A Segawa K Aoki N Matsuda M. Visfatin is released from 3T3-L1 adipocytes via a non-classical pathway. Biochem Biophys Res Commun. (2007) 359:194–201. 10.1016/j.bbrc.2007.05.096 17543285

[114] Garten A Petzold S Barnikol-Oettler A Korner A Thasler WE Kratzsch J. Nicotinamide phosphoribosyltransferase (NAMPT/PBEF/visfatin) is constitutively released from human hepatocytes. Biochem Biophys Res Commun. (2010) 391:376–81. 10.1016/j.bbrc.2009.11.066 19912992

[115] Friebe D Neef M Kratzsch J Erbs S Dittrich K Garten A. Leucocytes are a major source of circulating nicotinamide phosphoribosyltransferase (NAMPT)/pre-B cell colony (PBEF)/visfatin linking obesity and inflammation in humans. Diabetologia. (2011) 54:1200–11. 10.1007/s00125-010-2042-z 21298414 PMC3071946

[116] Schilling E Wehrhahn J Klein C Raulien N Ceglarek U Hauschildt S. Inhibition of nicotinamide phosphoribosyltransferase modifies LPS-induced inflammatory responses of human monocytes. Innate Immun. (2012) 18:518–30. 10.1177/1753425911423853 21975728

[117] Pillai VB Sundaresan NR Kim G Samant S Moreno-Vinasco L Garcia JG. Nampt secreted from cardiomyocytes promotes development of cardiac hypertrophy and adverse ventricular remodeling. Am J Physiol Heart Circ Physiol. (2013) 304:H415–26. 10.1152/ajpheart.00468.2012 23203961 PMC3774498

[118] Grolla AA Torretta S Gnemmi I Amoruso A Orsomando G Gatti M. Nicotinamide phosphoribosyltransferase (NAMPT/PBEF/visfatin) is a tumoural cytokine released from melanoma. Pigment Cell Melanoma Res. (2015) 28:718–29. 10.1111/pcmr.12420 26358657

[119] Audrito V Manago A Zamporlini F Rulli E Gaudino F Madonna G. Extracellular nicotinamide phosphoribosyltransferase (eNAMPT) is a novel marker for patients with BRAF-mutated metastatic melanoma. Oncotarget. (2018) 9:18997–9005. 10.18632/oncotarget.24871 29721178 PMC5922372

[120] Yoshida M Satoh A Lin JB Mills KF Sasaki Y Rensing N. Extracellular vesicle-contained eNAMPT delays aging and extends lifespan in mice. Cell Metab. (2019) 30:329–42 e325. 10.1016/j.cmet.2019.05.015 31204283 PMC6687560

[121] Lu YB Chen CX Huang J Tian YX Xie X Yang P. Nicotinamide phosphoribosyltransferase secreted from microglia via exosome during ischemic injury. J Neurochem. (2019) 150:723–37. 10.1111/jnc.14811 31269239

[122] Yoon MJ Yoshida M Johnson S Takikawa A Usui I Tobe K. SIRT1-mediated eNAMPT secretion from adipose tissue regulates hypothalamic NAD+ and function in mice. Cell Metab. (2015) 21:706–17. 10.1016/j.cmet.2015.04.002 25921090 PMC4426056

[123] Piacente F Caffa I Ravera S Sociali G Passalacqua M Vellone VG. Nicotinic acid phosphoribosyltransferase regulates cancer cell metabolism, susceptibility to NAMPT inhibitors, and DNA repair. Cancer Res. (2017) 77:3857–69. 10.1158/0008-5472.CAN-16-3079 28507103

[124] Manago A Audrito V Mazzola F Sorci L Gaudino F Gizzi K. Extracellular nicotinate phosphoribosyltransferase binds Toll like receptor 4 and mediates inflammation. Nat Commun. (2019) 10:4116. 10.1038/s41467-019-12055-2 31511522 PMC6739309

[125] O'Brien T Oeh J Xiao Y Liang X Vanderbilt A Qin A. Supplementation of nicotinic acid with NAMPT inhibitors results in loss of *in vivo* efficacy in NAPRT1-deficient tumor models. Neoplasia. (2013) 15:1314–29. 10.1593/neo.131718 24403854 PMC3884523

[126] Shames DS Elkins K Walter K Holcomb T Du P Mohl D. Loss of NAPRT1 expression by tumor-specific promoter methylation provides a novel predictive biomarker for NAMPT inhibitors. Clin Cancer Res. (2013) 19:6912–23. 10.1158/1078-0432.CCR-13-1186 24097869

[127] Sun Z Lei H Zhang Z. Pre-B cell colony enhancing factor (PBEF), a cytokine with multiple physiological functions. Cytokine Growth Factor Rev. (2013) 24:433–42. 10.1016/j.cytogfr.2013.05.006 23787158 PMC3791181

[128] Iqbal J Zaidi M. TNF regulates cellular NAD+ metabolism in primary macrophages. Biochem Biophys Res Commun. (2006) 342:1312–8. 10.1016/j.bbrc.2006.02.109 16516847

[129] Gosset M Berenbaum F Salvat C Sautet A Pigenet A Tahiri K. Crucial role of visfatin/pre-B cell colony-enhancing factor in matrix degradation and prostaglandin E2 synthesis in chondrocytes: possible influence on osteoarthritis. Arthritis Rheum. (2008) 58:1399–409. 10.1002/art.23431 18438860

[130] Moschen AR Gerner RR Tilg H. Pre-B cell colony enhancing factor/NAMPT/visfatin in inflammation and obesity-related disorders. Curr Pharm Des. (2010) 16:1913–20. 10.2174/138161210791208947 20370672

[131] Li Y Zhang Y Dorweiler B Cui D Wang T Woo CW. Extracellular Nampt promotes macrophage survival via a nonenzymatic interleukin-6/STAT3 signaling mechanism. J Biol Chem. (2008) 283:34833–43. 10.1074/jbc.M805866200 18945671 PMC2596403

[132] Audrito V Serra S Brusa D Mazzola F Arruga F Vaisitti T. Extracellular nicotinamide phosphoribosyltransferase (NAMPT) promotes M2 macrophage polarization in chronic lymphocytic leukemia. Blood. (2015) 125:111–23. 10.1182/blood-2014-07-589069 25368373

[133] Moschen AR Kaser A Enrich B Mosheimer B Theurl M Niederegger H. Visfatin, an adipocytokine with proinflammatory and immunomodulating properties. J Immunol. (2007) 178:1748–58. 10.4049/jimmunol.178.3.1748 17237424

[134] Fan Y Meng S Wang Y Cao J Wang C. Visfatin/PBEF/Nampt induces EMMPRIN and MMP-9 production in macrophages via the NAMPT-MAPK (p38, ERK1/2)-NF-kappaB signaling pathway. Int J Mol Med. (2011) 27:607–15. 10.3892/ijmm.2011.621 21327328

[135] Chang YC Chang TJ Lee WJ Chuang LM. The relationship of visfatin/pre-B-cell colony-enhancing factor/nicotinamide phosphoribosyltransferase in adipose tissue with inflammation, insulin resistance, and plasma lipids. Metabolism. (2010) 59:93–9. 10.1016/j.metabol.2009.07.011 19765775

[136] Stephens JM Vidal-Puig AJ. An update on visfatin/pre-B cell colony-enhancing factor, an ubiquitously expressed, illusive cytokine that is regulated in obesity. Curr Opin Lipidol. (2006) 17:128–31. 10.1097/01.mol.0000217893.77746.4b 16531748

[137] Araki S Dobashi K Kubo K Kawagoe R Yamamoto Y Kawada Y. Plasma visfatin concentration as a surrogate marker for visceral fat accumulation in obese children. Obesity. (2008) 16:384–8. 10.1038/oby.2007.54 18239648

[138] Yoshino J Mills KF Yoon MJ Imai S. Nicotinamide mononucleotide, a key NAD(+) intermediate, treats the pathophysiology of diet- and age-induced diabetes in mice. Cell Metab. (2011) 14:528–36. 10.1016/j.cmet.2011.08.014 21982712 PMC3204926

[139] Adya R Tan BK Punn A Chen J Randeva HS. Visfatin induces human endothelial VEGF and MMP-2/9 production via MAPK and PI3K/Akt signalling pathways: novel insights into visfatin-induced angiogenesis. Cardiovasc Res. (2008) 78:356–65. 10.1093/cvr/cvm111 18093986

[140] Kim SR Bae YH Bae SK Choi KS Yoon KH Koo TH. Visfatin enhances ICAM-1 and VCAM-1 expression through ROS-dependent NF-kappaB activation in endothelial cells. Biochim Biophys Acta. (2008) 1783:886–95. 10.1016/j.bbamcr.2008.01.004 18241674

[141] Lovren F Pan Y Shukla PC Quan A Teoh H Szmitko PE. Visfatin activates eNOS via Akt and MAP kinases and improves endothelial cell function and angiogenesis *in vitro* and *in vivo*: translational implications for atherosclerosis. Am J Physiol Endocrinol Metab. (2009) 296:E1440–9. 10.1152/ajpendo.90780.2008 19351806

[142] Romacho T Azcutia V Vazquez-Bella M Matesanz N Cercas E Nevado J. Extracellular PBEF/NAMPT/visfatin activates pro-inflammatory signalling in human vascular smooth muscle cells through nicotinamide phosphoribosyltransferase activity. Diabetologia. (2009) 52:2455–63. 10.1007/s00125-009-1509-2 19727662

[143] Adya R Tan BK Chen J Randeva HS. Pre-B cell colony enhancing factor (PBEF)/visfatin induces secretion of MCP-1 in human endothelial cells: role in visfatin-induced angiogenesis. Atherosclerosis. (2009) 205:113–9. 10.1016/j.atherosclerosis.2008.11.024 19166999

[144] Bae YH Bae MK Kim SR Lee JH Wee HJ Bae SK. Upregulation of fibroblast growth factor-2 by visfatin that promotes endothelial angiogenesis. Biochem Biophys Res Commun. (2009) 379:206–11. 10.1016/j.bbrc.2008.12.042 19100714

[145] Venereau E Ceriotti C Bianchi ME. DAMPs from cell death to new life. Front Immunol. (2015) 6:422. 10.3389/fimmu.2015.00422 26347745 PMC4539554

[146] Travelli C Colombo G Mola S Genazzani AA Porta C. NAMPT: a pleiotropic modulator of monocytes and macrophages. Pharmacol Res. (2018) 135:25–36. 10.1016/j.phrs.2018.06.022 30031171

[147] Minhas PS Liu L Moon PK Joshi AU Dove C Mhatre S. Macrophage *de novo* NAD(+) synthesis specifies immune function in aging and inflammation. Nat Immunol. (2019) 20:50–63. 10.1038/s41590-018-0255-3 30478397 PMC6768398

[148] Skokowa J Lan D Thakur BK Wang F Gupta K Cario G. NAMPT is essential for the G-CSF-induced myeloid differentiation via a NAD(+)-sirtuin-1-dependent pathway. Nat Med. (2009) 15:151–8. 10.1038/nm.1913 19182797

[149] Travelli C Consonni FM Sangaletti S Storto M Morlacchi S Grolla AA Nicotinamide phosphoribosyltransferase (NAMPT) acts as a metabolic gate for mobilization of myeloid-derived suppressor cells. Cancer Res. (2019) 79:1938–51. 10.1158/0008-5472.CAN-18-1544 30777853

[150] Weidemann A Johnson RS. Biology of HIF-1alpha. Cell Death Differ. (2008) 15:621–7. 10.1038/cdd.2008.12 18259201

[151] Gleyzer N Scarpulla RC. PGC-1-related coactivator (PRC), a sensor of metabolic stress, orchestrates a redox-sensitive program of inflammatory gene expression. J Biol Chem. (2011) 286:39715–25. 10.1074/jbc.M111.291575 21937425 PMC3220587

[152] Liu TF Vachharajani VT Yoza BK McCall CE. NAD+-dependent sirtuin 1 and 6 proteins coordinate a switch from glucose to fatty acid oxidation during the acute inflammatory response. J Biol Chem. (2012) 287:25758–69. 10.1074/jbc.M112.362343 22700961 PMC3406663

[153] Fernandes CA Fievez L Neyrinck AM Delzenne NM Bureau F Vanbever R. Sirtuin inhibition attenuates the production of inflammatory cytokines in lipopolysaccharide-stimulated macrophages. Biochem Biophys Res Commun. (2012) 420:857–61. 10.1016/j.bbrc.2012.03.088 22469470

[154] Koch C Samareh B Morishima T Mir P Kanz L Zeidler C GM-CSF treatment is not effective in congenital neutropenia patients due to its inability to activate NAMPT signaling. Ann Hematol. (2017) 96:345–53. 10.1007/s00277-016-2894-5 27966038

[155] Molugu TR Oita RC Chawla U Camp SM Brown MF Garcia GN (2020) Nicotinamide phosphoribosyltransferase purification using SUMO expression system. Anal Biochem, 113597 10.1016/j.ab.2020.113597 31982408 PMC7735423

[156] Yang K Lauritzen KH Olsen MB Dahl TB Ranheim T Ahmed MS. Low cellular NAD(+) compromises lipopolysaccharide-induced inflammatory responses via inhibiting TLR4 signal transduction in human monocytes. J Immunol. (2019) 203:1598–608. 10.4049/jimmunol.1801382 31427442

[157] Chang YH Chang DM Lin KC Shin SJ Lee YJ. Visfatin in overweight/obesity, type 2 diabetes mellitus, insulin resistance, metabolic syndrome and cardiovascular diseases: a meta-analysis and systemic review. Diabetes Metab Res Rev. (2011) 27:515–27. 10.1002/dmrr.1201 21484978

[158] Romacho T Sanchez-Ferrer CF Peiro C. Visfatin/Nampt: an adipokine with cardiovascular impact. Mediators Inflamm. (2013) 2013:946427. 10.1155/2013/946427 23843684 PMC3697395

[159] Chen X Zhao S Song Y Shi Y Leak RK Cao G. The role of nicotinamide phosphoribosyltransferase in cerebral ischemia. Curr Top Med Chem. (2015) 15:2211–2221. 10.2174/1568026615666150610142234 26059356 PMC5644507

[160] Wang P Miao CY. NAMPT as a therapeutic target against stroke. Trends Pharmacol Sci. (2015) 36:891–905. 10.1016/j.tips.2015.08.012 26538317

[161] Malyszko J Malyszko JS Mysliwiec M. Visfatin, a new adipocytokine, is predominantly related to inflammation/endothelial damage in kidney allograft recipients. TransplProc. (2009) 41:150–3. 10.1016/j.transproceed.2008.10.086 19249500

[162] Dambala K Vavilis D Bili E Goulis DG Tarlatzis BC. Serum visfatin, vascular endothelial growth factor and matrix metalloproteinase-9 in women with polycystic ovary syndrome. Gynecol Endocrinol. (2017) 33:529–33. 10.1080/09513590.2017.1296425 28300464

[163] Porter B Babbar S Ye SQ Maulik D. The role of nicotinamide phosphoribosyltransferase in pregnancy: a review. Am J Perinatol. (2016) 33:1327–36. 10.1055/s-0036-1582448 27135957

[164] Dahl TB Yndestad A Skjelland M Oie E Dahl A Michelsen A. Increased expression of visfatin in macrophages of human unstable carotid and coronary atherosclerosis: possible role in inflammation and plaque destabilization. Circulation. (2007) 115:972–80. 10.1161/CIRCULATIONAHA.106.665893 17283255

[165] Ye SQ Simon BA Maloney JP Zambelli-Weiner A Gao L Grant A. Pre-B-cell colony-enhancing factor as a potential novel biomarker in acute lung injury. Am J Respir Crit Care Med. (2005) 171:361–70. 10.1164/rccm.200404-563OC 15579727

[166] Moreno-Vinasco L Quijada H Sammani S Siegler J Letsiou E Deaton R. Nicotinamide phosphoribosyltransferase inhibitor is a novel therapeutic candidate in murine models of inflammatory lung injury. Am J Respir Cell Mol Biol. (2014) 51:223–8. 10.1165/rcmb.2012-0519OC 24588101 PMC4148034

[167] Jia SH Li Y Parodo J Kapus A Fan L Rotstein OD. Pre-B cell colony-enhancing factor inhibits neutrophil apoptosis in experimental inflammation and clinical sepsis. J Clin Invest. (2004) 113:1318–27. 10.1172/JCI19930 15124023 PMC398427

[168] Karampela I Christodoulatos GS Kandri E Antonakos G Vogiatzakis E Dimopoulos G. Circulating eNampt and resistin as a proinflammatory duet predicting independently mortality in critically ill patients with sepsis: a prospective observational study. Cytokine. (2019) 119:62–70. 10.1016/j.cyto.2019.03.002 30884428

[169] Ognjanovic S Bryant-Greenwood GD. Pre-B-cell colony-enhancing factor, a novel cytokine of human fetal membranes. Am J Obstet Gynecol. (2002) 187:1051–8. 10.1067/mob.2002.126295 12389004

[170] Ismail SA Mohamed SA. Serum levels of visfatin and omentin-1 in patients with psoriasis and their relation to disease severity. Br J Dermatol. (2012) 167:436–9. 10.1111/j.1365-2133.2012.10980.x 22486212

[171] Franco-Trepat E Alonso-Perez A Guillan-Fresco M Jorge-Mora A Gualillo O Gomez-Reino JJ. Visfatin as a therapeutic target for rheumatoid arthritis. Expert Opin Ther Targets. (2019) 23:607–18. 10.1080/14728222.2019.1617274 31074669

[172] Neubauer K Bednarz-Misa I Walecka-Zacharska E Wierzbicki J Agrawal A Gamian A. Oversecretion and overexpression of nicotinamide phosphoribosyltransferase/Pre-B colony-enhancing factor/visfatin in inflammatory bowel disease reflects the disease activity, severity of inflammatory response and hypoxia. Int J Mol Sci. (2019) 20:166. 10.3390/ijms20010166 30621173 PMC6337260

[173] Sayers SR Beavil RL Fine NHF Huang GC Choudhary P Pacholarz KJ. Structure-functional changes in eNAMPT at high concentrations mediate mouse and human beta cell dysfunction in type 2 diabetes. Diabetologia. (2019). 63:313–23. 10.1007/s00125-019-05029-y 31732790 PMC6946736

[174] Nielsen KN Peics J Ma T Karavaeva I Dall M Chubanava S. NAMPT-mediated NAD(+) biosynthesis is indispensable for adipose tissue plasticity and development of obesity. Mol Metab. (2018) 11:178–88. 10.1016/j.molmet.2018.02.014 29551635 PMC6001355

[175] Wang SN Miao CY. Targeting NAMPT as a therapeutic strategy against stroke. Stroke Vasc Neurol. (2019) 4:83–9. 10.1136/svn-2018-000199 31338216 PMC6613878

[176] Busso N Karababa M Nobile M Rolaz A Van Gool F Galli M. Pharmacological inhibition of nicotinamide phosphoribosyltransferase/visfatin enzymatic activity identifies a new inflammatory pathway linked to NAD. PLoS ONE. (2008) 3:e2267. 10.1371/journal.pone.0002267 18493620 PMC2377336

[177] Gerner RR Klepsch V Macheiner S Arnhard K Adolph TE Grander C. NAD metabolism fuels human and mouse intestinal inflammation. Gut. (2018) 67:1813–1823. 10.1136/gutjnl-2017-314241 28877980 PMC6145287

[178] Dalamaga M Christodoulatos GS Mantzoros CS. The role of extracellular and intracellular Nicotinamide phosphoribosyl-transferase in cancer: diagnostic and therapeutic perspectives and challenges. Metabolism. (2018) 82:72–87. 10.1016/j.metabol.2018.01.001 29330025

[179] Yaku K Okabe K Hikosaka K Nakagawa T. NAD metabolism in cancer therapeutics. Front Oncol. (2018) 8:622. 10.3389/fonc.2018.00622 30631755 PMC6315198

[180] Li XQ Lei J Mao LH Wang QL Xu F Ran T. NAMPT and NAPRT, key enzymes in NAD salvage synthesis pathway, are of negative prognostic value in colorectal cancer. Front Oncol. (2019) 9:736. 10.3389/fonc.2019.00736 31448236 PMC6691178

[181] Nacarelli T Lau L Fukumoto T Zundell J Fatkhutdinov N Wu S. NAD(+) metabolism governs the proinflammatory senescence-associated secretome. Nat Cell Biol. (2019) 21:397–407. 10.1038/s41556-019-0287-4 30778219 PMC6448588

[182] Fons NR Sundaram RK Breuer GA Peng S McLean RL Kalathil AN. PPM1D mutations silence NAPRT gene expression and confer NAMPT inhibitor sensitivity in glioma. Nat Commun. (2019) 10:3790. 10.1038/s41467-019-11732-6 31439867 PMC6706443

[183] Watson M Roulston A Belec L Billot X Marcellus R Bedard D. The small molecule GMX1778 is a potent inhibitor of NAD+ biosynthesis: strategy for enhanced therapy in nicotinic acid phosphoribosyltransferase 1-deficient tumors. Mol Cell Biol. (2009) 29:5872–5888. 10.1128/MCB.00112-09 19703994 PMC2772749

[184] Olesen UH Hastrup N Sehested M. Expression patterns of nicotinamide phosphoribosyltransferase and nicotinic acid phosphoribosyltransferase in human malignant lymphomas. APMIS. (2011) 119:296–303. 10.1111/j.1600-0463.2011.02733.x 21492230

[185] Cerna D Li H Flaherty S Takebe N Coleman CN Yoo SS. Inhibition of nicotinamide phosphoribosyltransferase (NAMPT) activity by small molecule GMX1778 regulates reactive oxygen species (ROS)-mediated cytotoxicity in a p53- and nicotinic acid phosphoribosyltransferase1 (NAPRT1)-dependent manner. J Biol Chem. (2012) 287:22408–17. 10.1074/jbc.M112.357301 22570471 PMC3381200

[186] Hasmann M Schemainda I. FK866, a highly specific noncompetitive inhibitor of nicotinamide phosphoribosyltransferase, represents a novel mechanism for induction of tumor cell apoptosis. Cancer Res. (2003) 63:7436–42. 14612543

[187] Olesen UH Christensen MK Bjorkling F Jaattela M Jensen PB Sehested M. Anticancer agent CHS-828 inhibits cellular synthesis of NAD. Biochem Biophys Res Commun. (2008) 367:799–804. 10.1016/j.bbrc.2008.01.019 18201551

[188] Galli U Travelli C Massarotti A Fakhfouri G Rahimian R Tron GC. Medicinal chemistry of nicotinamide phosphoribosyltransferase (NAMPT) inhibitors. J Med Chem. (2013) 56:6279–96. 10.1021/jm4001049 23679915

[189] Abu Aboud O Chen CH Senapedis W Baloglu E Argueta C Weiss RH. Dual and specific inhibition of NAMPT and PAK4 By KPT-9274 decreases kidney cancer growth. Mol Cancer Ther. (2016) 15:2119–29. 10.1158/1535-7163.MCT-16-0197 27390344 PMC5010932

[190] Sampath D Zabka TS Misner DL O'Brien T Dragovich PS. Inhibition of nicotinamide phosphoribosyltransferase (NAMPT) as a therapeutic strategy in cancer. Pharmacol Ther. (2015) 151:16–31. 10.1016/j.pharmthera.2015.02.004 25709099

[191] Montecucco F Cea M Bauer I Soncini D Caffa I Lasiglie D. Nicotinamide phosphoribosyltransferase (NAMPT) inhibitors as therapeutics: rationales, controversies, clinical experience. Curr Drug Targets. (2013) 14:637–43. 10.2174/1389450111314060003 23531116

[192] Chen H Wang S Zhang H Nice EC Huang C. Nicotinamide phosphoribosyltransferase (Nampt) in carcinogenesis: new clinical opportunities. Expert Rev Anticancer Ther. (2016) 16:827–38. 10.1080/14737140.2016.1190649 27186719

[193] Oita RC Camp SM Ma W Ceco E Harbeck M Singleton P. Novel mechanism for nicotinamide phosphoribosyltransferase inhibition of TNF-alpha-mediated apoptosis in human lung endothelial cells. Am J Respir Cell Mol Biol. (2018) 59:36–44. 10.1165/rcmb.2017-0155OC 29337590 PMC6039874

